# Molecular Diagnostics for WHO Priority Bacterial Pathogens: A Bibliometric Mapping of Diagnostic Platforms, Resistance Markers, and Antimicrobial Resistance Research Trends

**DOI:** 10.1002/mbo3.70394

**Published:** 2026-08-31

**Authors:** Selahattin Ünlü

**Affiliations:** ^1^ Department of Medical Microbiology, Faculty of Medicine Afyonkarahisar Health Sciences University Afyonkarahisar Turkey

**Keywords:** antimicrobial resistance, bibliometrics, molecular diagnostics, resistance markers, WHO priority pathogens, whole‐genome sequencing

## Abstract

Antimicrobial resistance (AMR) constrains effective treatment and carries implications for infection control, surveillance, and public health. The World Health Organization (WHO) priority bacterial pathogen framework has intensified the need for diagnostic innovation by redefining research priorities around organisms combining high disease burden with complex resistance profiles. Molecular diagnostics have accordingly moved beyond culture‐based workflows, integrating rapid pathogen identification, resistance‐marker detection, genomic surveillance, and clinical decision support. The present study conducted a bibliometric mapping of the literature on WHO priority pathogens. Rather than addressing resistance at a general level or a single pathogen or technology, it integrates priority pathogens, molecular platforms, and resistance markers within a single framework, tracing their joint thematic and temporal evolution along an explicit pathogen–platform–marker axis. Scopus‐indexed articles and reviews (2000–2025) were retrieved, yielding 1746 publications after screening adapted from the Preferred Reporting Items for Systematic Reviews and Meta‐Analyses (PRISMA) guidelines. Analyses used Bibliometrix/Biblioshiny, R, and VOSviewer. The literature expanded markedly after 2018, led by China and the United States. Methicillin‐resistant *Staphylococcus aureus* (MRSA), *Mycobacterium tuberculosis*, *Enterococcus faecium*, and the *Enterobacterales*–carbapenemase axis constituted the principal thematic cores, whereas conventional polymerase chain reaction (PCR)/nucleic acid amplification testing (NAAT) and whole‐genome sequencing were the dominant platforms. Overall, the field has evolved from pathogen detection into an AMR‐centered translational domain encompassing resistance prediction, genomic epidemiology, surveillance, and clinical decision support. Diagnostic development, stewardship, and surveillance depend on hybrid workflows coupling rapid marker‐targeted assays with genome‐based characterization, delivering actionable resistance within clinically meaningful timeframes, and extending coverage to underrepresented pathogens and platforms.

## Graphical abstract

This bibliometric mapping of 1746 publications (2000–2025) charts molecular diagnostics for WHO priority bacterial pathogens across platforms, targets, and resistance markers. Conventional PCR/NAAT and whole‐genome sequencing emerge as complementary core platforms, and the field evolves from targeted pathogen detection into an AMR‐centered translational domain spanning resistance prediction, genomic epidemiology, and surveillance.

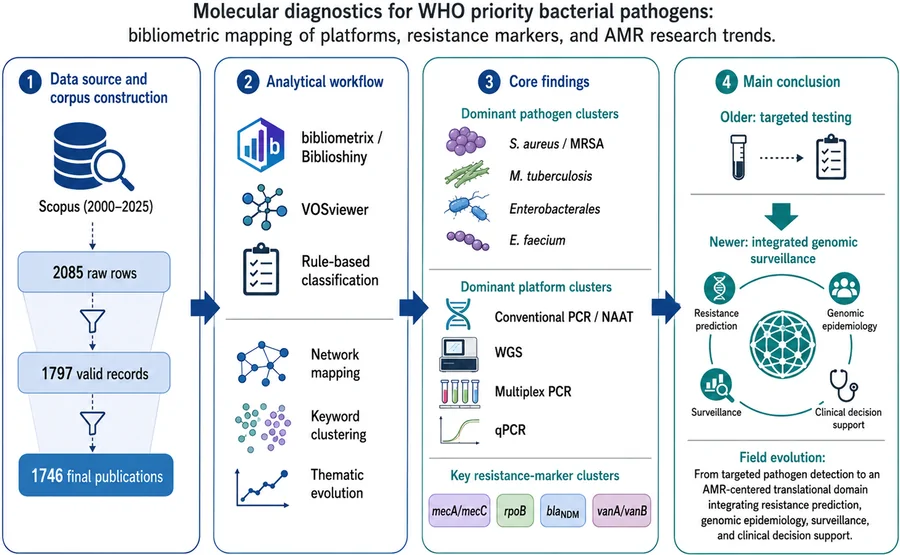

## 1 Introduction

Antimicrobial resistance (AMR) is one of the primary threats to global public health. In 2019, the number of deaths associated with bacterial AMR was estimated at 4.95 million, while the number of deaths directly attributable to AMR was estimated at 1.27 million. This burden is particularly evident in lower respiratory tract infections, bloodstream infections, and intra‐abdominal infections (Murray et al. 2022). The clinical and public health significance of AMR stems not only from its limitation of effective treatment options but also from the additional burden it places on healthcare systems due to increased mortality, the need for inappropriate or delayed empirical treatment, and rising healthcare costs (Murray et al. 2022; Kaprou et al. 2021). Therefore, the early, accurate, and clinically informed detection of resistant bacteria is of fundamental importance for optimizing treatment in a timely manner—particularly in severe infections—and for strengthening antimicrobial stewardship practices (Kaprou et al. 2021; Lapin et al. 2023; Peri et al. 2024).

This necessity has been further emphasized by the World Health Organization's (WHO) prioritization framework. The prioritization document published by the WHO in 2017 classified resistant bacteria into critical, high, and medium priority groups to guide new antibiotic research and development investments; specifically highlighting carbapenem‐resistant Gram‐negative bacteria under the critical priority category, and methicillin‐resistant *Staphylococcus aureus* (MRSA) and vancomycin‐resistant *Enterococcus faecium* (VRE) within the high‐priority group (Tacconelli et al. 2018). The same framework also addressed tuberculosis as a key component of the antibacterial R&D agenda (Tacconelli et al. 2018).

The WHO's 2024 update expanded the framework to 24 pathogens spanning 15 bacterial families, and reprioritization was performed through a multicriteria decision analysis combining mortality, incidence, resistance trends, transmissibility, preventability, treatability, and the state of the antibacterial development pipeline (Sati et al. 2025). The critical tier comprised carbapenem‐resistant *Acinetobacter baumannii*, carbapenem‐resistant and third‐generation cephalosporin‐resistant *Enterobacterales*, and rifampicin‐resistant *Mycobacterium tuberculosis*. Carbapenem‐resistant *Pseudomonas aeruginosa*, by contrast, was moved from the critical to the high‐priority tier on the basis of resistance trends observed in at least one WHO region and its comparatively lower transmission capability (Sati et al. 2025). The high‐priority tier comprises fluoroquinolone‐resistant *Salmonella* Typhi and *Shigella* spp., VRE, fluoroquinolone‐resistant nontyphoidal *Salmonella*, *Neisseria gonorrhoeae*, and MRSA, while the medium tier incorporated pathogens absent from the 2017 list, including macrolide‐resistant group A streptococci and penicillin‐resistant group B streptococci (Sati et al. 2025). This restructuring indicates that pathogen prioritization functions not as a fixed ranking but as a dynamic framework updated in response to shifts in resistance epidemiology.

The same framework, through its preventability criterion, guides not only antibacterial research and development and diagnostic priorities but also preventive strategies, including vaccine development pipelines directed against these pathogens (Hetta et al. 2026). The burden of multidrug‐resistant healthcare‐associated infection is not, however, confined to the organisms named in prioritization lists; reports of multidrug‐resistant isolates of opportunistic *Enterobacterales* such as *Serratia marcescens* in intensive care settings indicate that the resistance problem extends beyond the most frequently studied WHO priority pathogens (AL‐Kadmy et al. 2025).

As drug‐resistant pathogens have gained greater prominence within the global prioritization framework, this has also accelerated the shift toward molecular approaches—which are faster, more sensitive, and clinically more actionable—over culture‐based and relatively slower methods for diagnosing AMR (Tacconelli et al. 2018; Sati et al. 2025). Targeted polymerase chain reaction (PCR), quantitative PCR (qPCR)/real‐time PCR, and multiplex PCR‐based platforms are widely used in clinical microbiology laboratories for the early detection of specific pathogens and resistance genes; this contributes to early treatment optimization, particularly in critical infections (Kaprou et al. 2021).

The clinical value of this diagnostic gain is realized to the extent that the result is translated into a treatment decision. A network meta‐analysis in bloodstream infection showed that rapid diagnostic tests, when deployed together with antimicrobial stewardship programmes, were associated with a significant reduction in mortality compared with blood culture alone (odds ratio 0.72; 95% CI, 0.59–0.87), and that this advantage was maintained against blood culture combined with stewardship (Peri et al. 2024). This indicates that the clinical impact of molecular diagnostics depends less on assay performance than on the speed at which results are reported and on the presence of a stewardship structure capable of acting on them (Lapin et al. 2023; Peri et al. 2024). In critical care, this linkage operates through earlier transition from empirical broad‐spectrum therapy to targeted treatment, reduction of unnecessary antibiotic exposure, and support for infection control decisions through early detection of resistant isolates (Alatawi et al. 2025). Genotypic resistance data, when fed into institutional antibiograms and regional surveillance systems, further provide a basis for updating empirical treatment policy (Alatawi et al. 2025). In parallel, whole‐genome sequencing (WGS) has gained increasing importance in clinical microbiology practice by enabling the assessment not only of resistance determinants but also of clonal relationships, spread dynamics, and genomic context (Rossen et al. 2018; Bagger et al. 2024). Metagenomic next‐generation sequencing (mNGS) enables the detection of a wide range of pathogens in a culture‐independent manner, while clustered regularly interspaced short palindromic repeats (CRISPR)‐based diagnostic systems stand out among next‐generation diagnostic tools due to their high specificity and rapid detection potential (Wang et al. 2022; Zhang et al. 2024).

However, the rapid expansion of this field has led to the literature becoming increasingly heterogeneous and thematically fragmented. Research clusters emerging around different pathogens, diagnostic platforms, resistance markers, and clinical application contexts make it difficult to fully grasp the field's holistic structure through traditional narrative reviews (Sun et al. 2022; Jangid et al. 2023; Shi 2025). In this context, bibliometric analysis offers a robust methodological framework for systematically mapping the voluminous and dispersed literature across dimensions of productivity, impact, international collaboration, conceptual clustering, and temporal evolution (Aria and Cuccurullo 2017). Indeed, bibliometric studies on antibiotic‐resistant bacteria, carbapenem‐resistant *A. baumannii*, and *S. aureus* drug resistance demonstrate that these research areas exhibit distinct thematic foci, evolving collaboration networks, and shifting research priorities over time (Sun et al. 2022; Jangid et al. 2023; Shi 2025).

Nevertheless, a significant portion of existing bibliometric studies either address the broad AMR literature at a general level or focus on specific subfields. For example, large‐scale bibliometric analyses have identified global publication patterns and key research axes regarding antibiotic‐resistant bacteria; in contrast, more recent studies have examined single‐pathogen areas such as carbapenem‐resistant *A. baumannii* or *S. aureus* antibiotic resistance separately (Sun et al. 2022; Jangid et al. 2023; Shi 2025). When the scope of these studies is evaluated collectively, it becomes evident that mapping approaches integrating WHO‐priority bacterial pathogens, molecular diagnostic platforms, resistance markers, and their temporal and thematic evolution within a single bibliometric framework remain relatively limited (Sun et al. 2022; Jangid et al. 2023; Shi 2025).

Bibliometric mapping and evidence‐synthesis methodologies address different classes of research questions. Systematic and scoping reviews are designed to answer bounded questions, such as the pooled diagnostic accuracy of a defined assay against a reference standard or the extent of available evidence for a specified intervention, and require studies that share a comparable outcome measure (Munn et al. 2018). The objectives of the present study are structural rather than effect‐oriented: to quantify productivity and impact, to resolve collaboration architecture, and to determine how pathogens, platforms and resistance markers cluster and shift across a 25‐year period. The corresponding corpus spans assay development, clinical validation, surveillance and genomic epidemiology, study types that share no common effect measure and cannot therefore be pooled or graded in the manner required by review methodologies (Munn et al. 2018; Donthu et al. 2021). Bibliometric mapping instead operates on the metadata of the literature itself—authorship, affiliation, citation, and keyword fields—and is accordingly suited to describing the structure and temporal trajectory of a field of this size and heterogeneity (Aria and Cuccurullo 2017; Donthu et al. 2021). The two approaches are complementary: by showing where evidence has concentrated and where it remains sparse, bibliometric mapping can help define the boundaries of subsequent systematic reviews (Donthu et al. 2021).

Building on this gap, the present study aims to bibliometrically map the molecular diagnostics literature in the context of WHO‐priority bacterial pathogens. The study comprehensively evaluated publication dynamics, core journals, leading authors/institutions/countries, patterns of international collaboration, dominant molecular platforms, frequently studied resistance markers, thematic clusters of keywords, and the temporal evolution of the field. Thus, the study aims to reveal not only the quantitative growth of the molecular bacterial diagnostics literature but also its AMR‐focused translational orientation and conceptual restructuring along the pathogen–platform–marker axis.

## 2 Materials and Methods

This study is a descriptive and explanatory bibliometric research conducted to examine the production dynamics, collaboration patterns, conceptual clustering, and temporal evolution of the literature on molecular bacterial diagnostics and AMR. The methodological workflow consisted of data extraction from the Scopus database, multistage corpus construction adapted to the Preferred Reporting Items for Systematic Reviews and Meta‐Analyses (PRISMA) 2020 guidelines, technical data cleaning and metadata restructuring, network‐theme analyses based on Bibliometrix/Biblioshiny and VOSviewer, and study‐specific rule‐based text‐matching steps. All field‐specific bibliometric findings were reported based on the final Scopus corpus (*n* = 1746) created following the thematic suitability screening (Table 1).

**Table 1:** Data layers, software components, and analytic roles used in the study.

Component	Scope/version	Primary function	Output types
Scopus data source	2000–2025; article/review; English	Primary bibliographic record pool and subject‐area profile	Raw CSV; analyze aggregates
Cleaned bibliographic data set	*n* = 1797	General bibliometric profile and quality control	Total records, journals, authors, citations, and CAGR
Final analytical corpus	*n* = 1746	All main table and figure analyses	Networks, thematic maps, and platform/pathogen matrices
R 4.5.3 + Bibliometrix 5.3.0	Code‐based analysis environment	Performance, social structure, conceptual structure, and thematic evolution	Thematic map, thematic evolution, Figures 6 and 7
Biblioshiny	Bibliometrix GUI	Metadata appraisal, performance summaries, and interactive exploration	Preliminary outputs for the most relevant sources, words, and country collaborations
VOSviewer	Network visualization	Country coauthorship, keyword co‐occurrence, and overlay mapping	Network maps of the types shown in Figures 4, 5, and 8
Python‐based scripts	Customized visualization	PRISMA diagram, annual trends	Figures 1, 2, 3

Abbreviations: CAGR, compound annual growth rate; CSV, comma‐separated values; GUI, graphical user interface; PRISMA, Preferred Reporting Items for Systematic Reviews and Meta‐Analyses.

### 2.1 Study Design and Data Source

This study is a retrospective, single‐database bibliometric investigation based on secondary analysis of bibliographic metadata. The use of Scopus as the sole data source reflects an analytical requirement rather than convenience. Coauthorship, keyword co‐occurrence, and citation‐based analyses require author identifiers, affiliation and address fields, keyword field structures, and citation counts to reside within a single, internally consistent metadata schema. When records from different databases are merged, the same document may carry divergent citation counts, different author identifier systems, and different keyword field definitions (e.g., author and index keywords in Scopus vs. author keywords and KeyWords Plus in Web of Science); harmonizing these fields and de‐duplicating records can introduce more uncertainty than it removes. PubMed provides neither cited‐reference nor citation data and is therefore unsuited to the computation of citation‐based indicators (total citations, mean citations, *h*‐index, and co‐citation networks). Dimensions applies a different indexing depth that encompasses preprints and non‐peer‐reviewed content, which would alter the definition of the corpus rather than extend it. Scopus offers broader journal coverage than the Web of Science Core Collection among biomedical and microbiological titles (Mongeon and Paul‐Hus 2016; Singh et al. 2021), provides structured author and affiliation identifiers, and its export format is supported directly and at full metadata level by Bibliometrix/Biblioshiny and VOSviewer (Aria and Cuccurullo 2017). Scopus was searched on April 14, 2026. The search was limited to journal publications indexed as articles or reviews, published in English, and dated between 2000 and 2025. No subject‐area filter was applied at the retrieval stage to avoid the premature exclusion of potentially relevant studies from adjacent translational, microbiological, and molecular research domains. Because the study did not involve human participants, animals, biological specimens, or identifiable personal data, the analysis was conducted entirely on publicly available bibliographic records.

The retrieved records were exported in CSV format for technical cleaning, screening, and bibliometric analysis, while the original database field structure was preserved as far as possible. This approach enabled the direct transfer of fields such as title, authors, publication year, journal title, abstract, keywords, citations, affiliations, and author identifiers into the Bibliometrix/Biblioshiny and VOSviewer workflows. For subject‐area distribution, aggregated subject‐area assignments obtained from the Scopus Analyze interface were used.

### 2.2 Search Strategy and Conceptual Query Architecture

The search strategy was iteratively developed to encompass the dimensions of clinical microbiology, molecular diagnostics, and AMR within the subject area. The query architecture is structured around three main blocks: (i) molecular diagnostics platforms and nucleic acid‐based methods; (ii) WHO‐priority bacterial pathogens and clinically relevant pathogens/pathogen families; (iii) AMR, resistance markers, and resistance targets. At the application level, platform terms such as PCR, qPCR/real‐time PCR, multiplex PCR, digital PCR, WGS, metagenomic sequencing, nanopore sequencing, CRISPR‐based diagnostics, loop‐mediated isothermal amplification (LAMP)/isothermal amplification, and point‐of‐care (POC) molecular systems were included. These terms were evaluated in conjunction with target pathogens, such as *S. aureus, E. faecium, M. tuberculosis*, *Enterobacterales*, *P. aeruginosa*, and *A. baumannii*.

The query development process was conducted in layers; initially, broader platform and biomarker blocks were tested, and subsequently, a narrowed‐down Scopus set with the highest analytical applicability and thematic specificity was selected as the foundation for the final corpus. This approach aimed to preserve the clinical and translational core of the field while maintaining the manageability of the data volume. The complete Scopus search query is provided in Table S1.

### 2.3 Inclusion and Exclusion Criteria and PRISMA‐Based Corpus Construction

**Figure 1:**
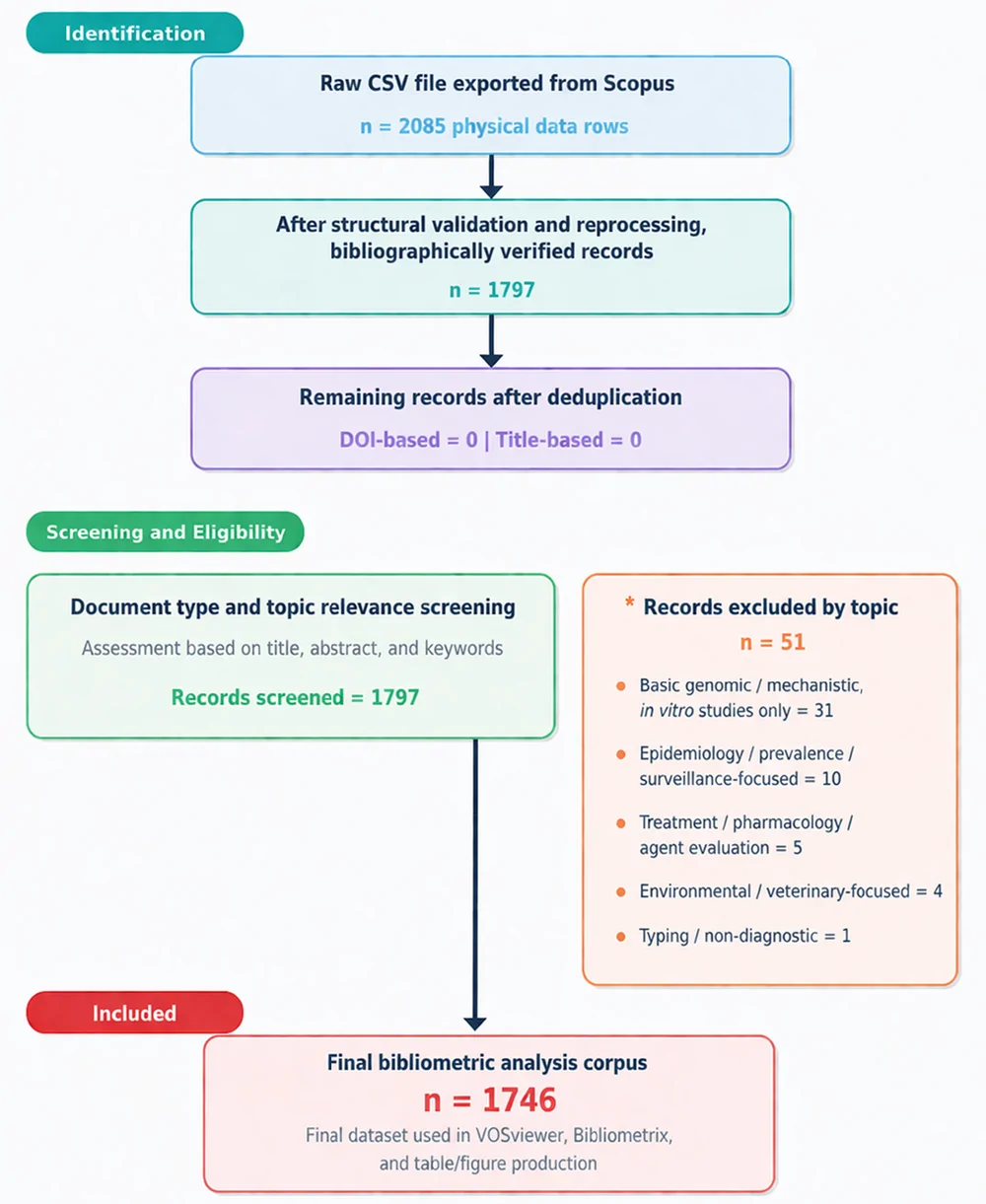
PRISMA‐adapted record selection and corpus construction flow diagram. *Although certain records may have overlapped across multiple exclusion categories, the final exclusion reason was assigned to the primary category identified for each record. DOI, digital object identifier; PRISMA, Preferred Reporting Items for Systematic Reviews and Meta‐Analyses.

The record selection and screening process followed a PRISMA‐adapted multistage workflow comprising technical restructuring, eligibility screening, and final corpus construction (Figure 1). Inclusion criteria were defined as publication between 2000 and 2025; indexing in Scopus as an article or review; publication in English; and a clear focus, at the level of the title, abstract, or keywords, on bacterial molecular diagnostics, resistance‐marker detection, diagnostic platform evaluation, pathogen identification, or clinically relevant translational applications related to AMR. Exclusion criteria included studies focused primarily on basic genomics or mechanistic biology without a diagnostic application, prevalence or surveillance studies without a molecular diagnostic emphasis, treatment‐ or pharmacology‐centered publications, studies in environmental or veterinary contexts, and records limited to nondiagnostic typing or phylogeny.

The raw Scopus export contained 2085 physical data rows. Because some records were fragmented across multiple lines in the abstract, affiliation, or related metadata fields, a technical restructuring step was first performed to restore field integrity and reconstruct bibliographically valid records. This step yielded 1797 records suitable for conceptual screening. Subject‐relevance screening was then conducted by the author at the title, abstract, and keyword levels using prespecified eligibility rules aligned with the study's diagnostic and translational scope. Borderline records were not automatically excluded; instead, they were reassessed conservatively against the predefined criteria and retained when meaningful diagnostic relevance could be justified based on the available bibliographic fields. To enhance transparency, the inclusion and exclusion process was documented through an explicit exclusion list generated during screening. This conceptual screening resulted in the exclusion of 51 records, yielding a final analytical corpus of 1746 publications.

### 2.4 Software Environment, Data Import, and Metadata Appraisal

All analyses were conducted within a multisoftware yet methodologically integrated ecosystem. The Bibliometrix package (version 5.3.0) in R (version 4.5.3) served as the core analytical backbone; Biblioshiny, the package's graphical user interface, was used for metadata appraisal, performance analysis, and interactive exploration. Scopus CSV files were first converted into a Bibliometrix‐compatible bibliographic data framework, and then the completeness of the title, author, journal title, abstract, institution/address, and reference fields was assessed using the metadata appraisal outputs in Biblioshiny. This review facilitated consideration of metadata gaps in specific fields during the interpretation phase, particularly in conceptual and social‐structure analyses.

VOSviewer was used to visualize cross‐national coauthorship networks, keyword co‐occurrence, and overlay visualizations. Python‐based scripts were used to generate a PRISMA flow diagram, annual trend graphs, and customized visualizations for the country production map.

### 2.5 Data Cleaning, Metadata Restructuring, and Standardization

Data preprocessing was conducted at two levels: technical validation and analytical standardization. During the technical validation phase, line shifts caused by multiline metadata fields were corrected; the title, year, citation count, source title, author fields, and institution/address information were realigned. During the analytical standardization phase, author identifiers, institution/country names, and keyword variants were harmonized. The total number of authors was calculated using unique identifiers derived from the Scopus “Author(s) ID” field; productivity at the institutional and country levels was determined through rule‐based parsing of the “Affiliations” field. In this process, variants such as USA/United States of America, UK/United Kingdom, and Republic of Korea/South Korea were standardized to a single form.

In conceptual structure analyses, the “Author's Keywords (DE)” field was primarily used to minimize the ambiguity caused by the interpretation of general index terms automatically generated by the database. However, since the same concept may appear in different orthographic forms, semantic equivalence was taken into account during the analyses. Thesaurus‐based standardization was applied to reduce terminological fragmentation in keyword networks and thematic map/cluster analyses.

### 2.6 Bibliometric Analysis Strategy

The analytical workflow was structured along three axes: performance analysis, social‐structure analysis, and conceptual structure analysis. Within the scope of performance analysis, annual scientific output, annual growth rate, journal and author productivity, total citations, average citations per document, average document age, and the rate of international coauthorship were evaluated.

In the social‐structure analysis, the cross‐country coauthorship network was evaluated. For this purpose, country labels were derived from the institution/address fields, and a country‐level coauthorship matrix was then constructed. To enhance interpretability in network visualization, low‐frequency nodes were filtered using a conservative threshold, and node sizes were interpreted to reflect the relative weight of collaboration among countries, while edge thicknesses reflected the intensity of coauthorship.

As part of the conceptual structure analysis, outputs including the most frequent keywords, keyword co‐occurrence network, trending topics, word frequency over time, thematic map, and thematic change/evolution were generated. The keyword co‐occurrence network was modeled using the co‐occurrence approach in Bibliometrix/Biblioshiny and VOSviewer, based on the DE domain. Network edge weights were scaled using association normalization logic; force‐directed layout strategies were adopted for visual arrangement, and only the most relevant nodes were visualized to preserve interpretability (Table 2).

**Table 2:** Bibliometric modules used and their analytical purposes.

Analytical module	Generated output(s)	Analytical purpose
Performance analysis	Annual scientific output, growth rate, source and author productivity, citation metrics	To determine the volume, visibility, and growth trends of the literature
Source analysis	Most Relevant Sources, journal distributions, time series	To identify core journals and publication concentration
Social‐structure analysis	Country Collaboration Network, Country Productivity Map	To reveal patterns of inter‐country coauthorship and collaboration hubs
Conceptual structure analysis	Keyword co‐occurrence network (DE field, association normalization), overlay visualization	To identify thematic clusters, relationships between concepts, and temporal shifts
Thematic structure analysis	Thematic map	To interpret the location of themes based on levels of centrality and density
Thematic evolution analysis	Thematic evolution map (2000–2010, 2011–2020, and 2021–2025)	To track conceptual continuity and period‐specific restructuring
Study‐specific derived analyses	Pathogen‐platform heat map, pathogen‐marker matrix, and WHO priority clusters	To visualize field‐specific translational patterns and resistance axes

Abbreviation: WHO, World Health Organization.

### 2.7 Thematic Map, Thematic Evolution, and Overlay Analysis

Thematic map analysis was conducted using the DE field, and clusters were evaluated along the centrality and density axes. Within this framework, themes were classified into motor, niche, core, and emerging/declining themes. The position of themes was interpreted as reflecting both their relationship to the field's general literature and their level of development within the cluster. The thematic evolution analysis, on the other hand, was divided into three time windows to track long‐term conceptual continuity: 2000–2010, 2011–2020, and 2021–2025. This periodization was chosen to highlight the trajectory of change extending from marker‐ and PCR‐focused structures in the early period, through genomic and resistance axes in the middle period, to an integrated AMR perspective. Overlay visualizations were created in VOSviewer using a color scale based on the average publication year, thereby ensuring the differentiation between early‐ and recent‐period themes.

### 2.8 Rule‐Based Text Matching, Derived Variables, and Internal Validation

A portion of the tables and figures was based on rule‐based text‐matching procedures applied to bibliographic metadata. For this purpose, the title, abstract, author‐keyword, and index‐keyword fields were combined into a unified text corpus, and predefined dictionary‐based rules were used to assign categories for pathogen identity, molecular platform, resistance marker/target, and clinical context. Pathogen classification was organized in alignment with WHO priority groups (critical, high, and medium), while platform classification comprised the following categories: conventional PCR/nucleic acid amplification testing (NAAT), WGS, multiplex PCR, qPCR/real‐time PCR, nanopore sequencing, metagenomic sequencing, CRISPR‐based diagnostics, digital PCR, LAMP/isothermal amplification, and POC molecular systems.

For resistance markers, the monitored targets included mecA/mecC, vanA/vanB, *blaKPC, blaNDM, blaOXA‐48, blaVIM, blaIMP, mcr, rpoB, katG, inhA, gyrA, parC, and 16S rRNA*. Clinical‐context coding was organized into top‐level categories, including bacteremia/sepsis, pneumonia/respiratory tract infection, tuberculosis, urinary tract infection, gonorrhea/sexually transmitted infection, hospital/intensive care unit infection, and enteric/foodborne infection. Thematic clusters were likewise assigned interpretive labels based on dominant keyword patterns identified across the final corpus.

To reduce artificial fragmentation and improve consistency across downstream analyses, the rule‐based framework was supported by thesaurus‐guided harmonization. Separate thesaurus files were used for country‐name normalization and keyword standardization, including consolidating synonymous or variant pathogen‐related and *Enterobacterales*‐related expressions into unified analytical labels. This standardization step was intended to improve the interpretability of co‐occurrence, thematic, and collaboration maps and to reduce noise arising from orthographic or indexing variation.

To strengthen methodological transparency, the study incorporated two complementary quality‐control layers. First, corpus construction was supported by an explicit title/abstract/keyword‐based relevance screening process, through which records were retained or excluded according to predefined diagnostic and translational eligibility criteria. Second, because several analytical variables in the final corpus were derived through rule‐based text matching, a separate post‐classification validation step was applied to a random subset of records to assess the face validity of the automated pathogen‐, platform‐, marker‐, and clinical‐context assignments. This two‐stage approach was intended to distinguish corpus‐level eligibility control from variable‐level classification control, thereby improving the internal consistency of the bibliometric framework.

To improve the internal validity of the derived variables, a validation subset was constructed from the final analytical corpus. Using a fixed random seed, 150 records were randomly selected from the screened data set (*n* = 1746) and reexamined at the title, abstract, author‐keyword, and index‐keyword levels to assess the face validity of the rule‐based assignments for pathogen category, molecular platform, resistance marker/target, and clinical context. Domain‐level concordance was defined as the presence of at least one manually supportable matching label within the automated assignment for the respective domain. The validation subset showed concordance rates of 91.3% for pathogen labels, 100.0% for platform labels, 100.0% for resistance‐marker labels, and 86.7% for clinical‐context labels, corresponding to an overall average agreement of 94.5%. Most discordant cases were related to broad multitopic abstracts, overlapping terminology, or context‐dependent mentions that did not reflect the primary analytical focus of the publication. The construction of the rule‐based classifications and the screening of the final corpus were carried out by a single researcher. To reduce record‐level error, the 150‐record validation subset was reassessed in a separate step, with the original rule‐based outputs concealed and after a defined time interval following the initial screening; the agreement rates reported above are based on this blinded reassessment.

### 2.9 Visualization, Interpretation, and Reproducibility

Bibliometric outputs were evaluated not only at the frequency level but also in terms of relational structure and temporal evolution. In network graphs, node sizes were associated with relative importance or frequency, while links were associated with co‐occurrence or collaboration strength. Color clusters were used as visual identifiers representing thematic or social subcommunities. During the interpretation phase, care was taken not to reduce the findings to individual term frequencies; each output was considered in the context of its production structure, collaboration patterns, and conceptual evolution.

In terms of reproducibility, the data source, software ecosystem, periodization logic, standardization rules, and core analytical modules have been clearly defined. When the same Scopus data set is imported into the Bibliometrix/Biblioshiny and VOSviewer environments, similar performance indicators, country collaboration structures, keyword networks, thematic maps, and thematic evolution patterns can be reproduced. However, the study adopts a controlled hybrid approach that combines bibliometric standardization with domain expertise rather than a fully automated machine learning pipeline; expert interpretation was incorporated, particularly in the steps of keyword harmonization and the naming of thematic clusters.

## 3 Results

### 3.1 Corpus Creation, General Profile, and Growth Dynamics

The overall bibliometric profile showed that the final analytical corpus for the 2000–2025 period comprised 1746 records published across 432 journals, involving 11,277 unique authors and 2936 unique keywords. Multiauthored publications predominated within the data set. The corpus accumulated a total of 42,075 citations, corresponding to an average of 24.10 citations per document. In addition, the annual compound growth rate of 17.10% indicates a rapidly expanding research field (Table 3).

**Table 3:** General bibliometric characteristics of the publications included in the study.

Indicator	Value
Final analytical data set	1746
Time span	2000–2025
Total number of journals	432
Total number of authors	11,277
Number of single‐authored publications	18
Number of multiauthored publications	1728
Total number of keywords	2936
Average annual growth rate (%)	17.10
Total citations	42,075
Average citations per document	24.10

*Note:* The total number of authors refers to the number of unique authors derived from the Author(s) ID field; the total number of keywords refers to the number of unique keywords in the Author‐Keywords field. The average annual growth rate was calculated using the compound annual growth rate (CAGR) approach for the 2000–2025 period.

In addition, validation of a random subset of 150 records demonstrated high concordance between rule‐based and manually reviewed assignments, yielding an overall average agreement of 94.5% across the derived pathogen, platform, resistance‐marker, and clinical‐context variables.

**Figure 2:**
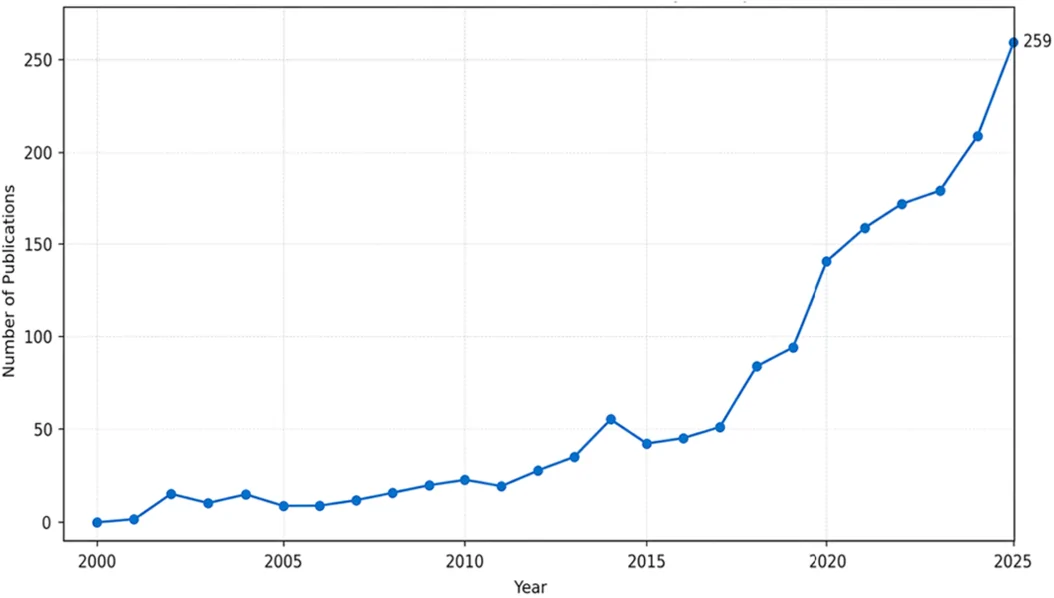
Annual publication distribution in the final Scopus corpus (*n* = 1746).

A distribution of publications by year shows that the field has accelerated rapidly, particularly after 2018. The annual number of publications followed a relatively limited and fluctuating trend during the 2000–2017 period; it was 84 in 2018, 95 in 2019, 143 in 2020, 157 in 2021, 169 in 2022, 176 in 2023, 208 in 2024, and 259 in 2025 (Figure 2). This pattern suggests that the literature on molecular bacterial diagnostics and AMR has exhibited a translational expansion, particularly accelerating in the post‐pandemic period.

**Figure 3:**
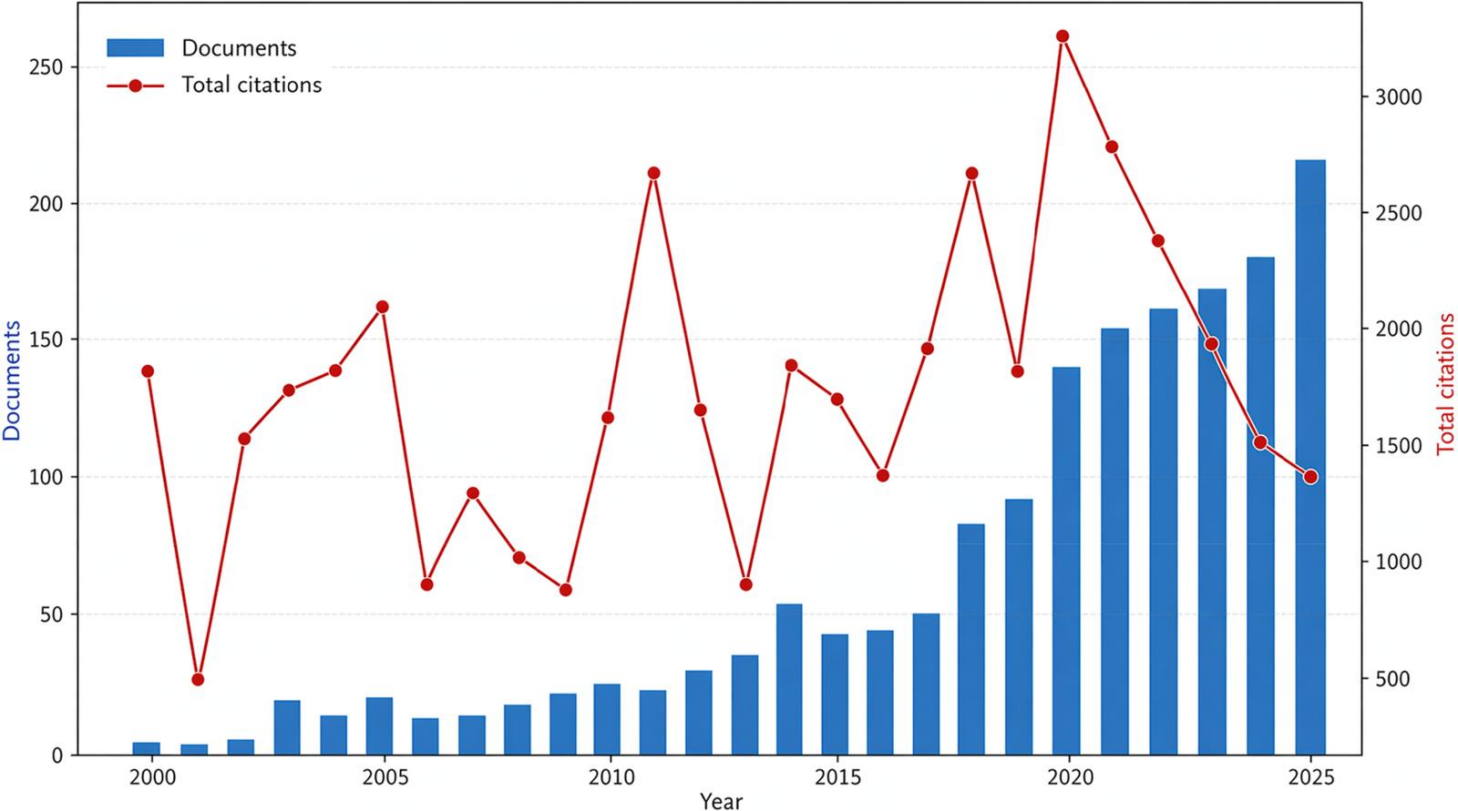
Annual publication output and total citation trend in the final corpus.

When publication output and citation impact are evaluated together, it becomes clear that the field is not only growing quantitatively but is also emerging as a center of increasing visibility and influence. When publication and citation trends are evaluated by year, the molecular diagnostics literature has gained strong growth momentum, particularly after 2018 and more notably starting in 2020, with the annual number of documents increasing steadily, while the total citation curve exhibits a more fluctuating pattern, and it is observed that this increase has not yet fully reflected in the citation load due to the shorter citation accumulation period of studies published in recent years (Figure 3).

The subject‐area distribution indicates that the field is primarily centered on clinical medicine and infectious microbiology, yet it produces a hybrid knowledge domain that strongly intersects with genomics, molecular biology, and pharmacology. 42.0% of subject‐area classifications fall under Medicine, 24.0% under Immunology and Microbiology, 14.6% under Biochemistry, Genetics and Molecular Biology, and 8.5% under Pharmacology, Toxicology and Pharmaceutics. The Agricultural and Biological Sciences, Environmental Science, and Multidisciplinary categories, which contribute at lower rates but remain significant, indicate that the field extends beyond clinical diagnosis to fundamental molecular mechanisms and translational biomarker studies.

### 3.2 Publication Outlets, Productive Actors, and Geographical Structure

An examination of the journal landscape reveals that publications cluster around certain core journals but do not exhibit intense centralization. Among the most productive and influential journals, the Journal of Clinical Microbiology, Frontiers in Microbiology, Antibiotics, Microbiology Spectrum, and the Journal of Global Antimicrobial Resistance stand out; the Journal of Clinical Microbiology has drawn attention as the primary publication venue in terms of both volume and impact, with 98 publications and 9202 total citations. The fact that Antimicrobial Agents and Chemotherapy demonstrates a high‐impact profile with 3271 total citations and an average of 86.08 citations per article, despite having only 38 publications indicates that historically established core journals in the field remain influential (Table S2). On the other hand, the significant rise in recent years of newer, open‐access platforms such as Frontiers in Microbiology, Antibiotics, and Microbiology Spectrum suggests that the publication ecosystem is shifting toward journals that circulate more rapidly and are visibility‐focused.

#### 3.2.1 Authors, Institutions, and Countries

Productivity patterns at the author and institutional levels indicate that the literature exhibits a multiactor structure with a limited degree of concentration. Among the most productive authors, Y. Yu and T. G. Clark stand out; names such as J. Parkhill, J. E. Phelan, and S. J. Peacock have drawn attention not only for their productivity but also for their impact, as evidenced by higher average citation counts and *h*‐index values (Table S3). Similarly, while the Zhejiang University School of Medicine and Islamic Azad University lead the list in terms of publication count, institutions such as the University of Cambridge, Statens Serum Institut, and the University of Copenhagen stand out for their higher total citation counts and average impact values (Table S4).

At the country level, China (263 publications), the United States (252 publications), the United Kingdom (130 publications), and Germany (127 publications) emerged as the primary production centers of the corpus. However, when total citations and international collaboration profiles are evaluated together, it is observed that countries such as the United States, the United Kingdom, Germany, and France occupy a central position in global knowledge circulation, particularly due to their higher Multiple Country Publications/Single Country Publications (MCP/SCP) ratios and higher average citation values. While countries like China and India exhibit lower MCP/SCP ratios despite high production volumes, the high international coauthorship rates of countries such as the United Kingdom (4.42) and France (2.08) indicate a higher degree of network centrality (Table S5).

**Figure 4:**
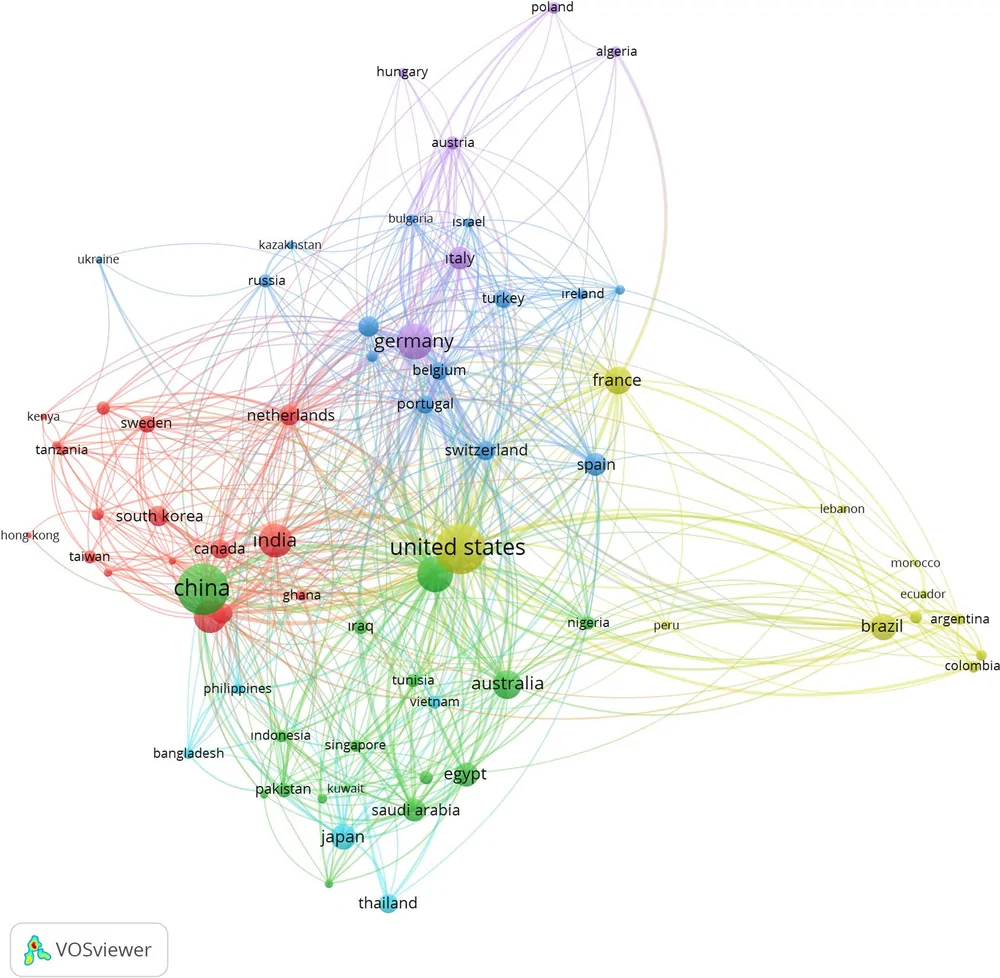
Country coauthorship network in the final Scopus corpus. Node size indicates publication volume, line thickness indicates collaboration strength, and colors denote network clusters.

The cross‐country coauthorship network revealed that the United States, China, and Germany emerged as the largest nodes with the highest connection density, while France, India, Australia, and Brazil served as bridges between different regional clusters (Figure 4).

### 3.3 High‐Impact Publications and Core Studies of the Knowledge Base

The top 20 most‐cited publications, listed in Table S6, demonstrate that the field initially took shape strongly around multiplex PCR, qPCR, and target‐specific resistance markers. Poirel and colleagues' multiplex PCR study on the detection of acquired carbapenemase genes ranked first, with 1762 citations and a normalized citation index of 117.47, followed by classic articles in the Journal of Clinical Microbiology and Antimicrobial Agents and Chemotherapy focusing on mec elements, MRSA subtypes, and staphylococcal resistance markers. The same publication set also demonstrates a historical shift toward tuberculosis resistance markers and WGS‐based studies; Seifert et al.'s systematic review of isoniazid resistance and Cohen et al.'s WGS‐based analysis of the evolution of extensively drug‐resistant tuberculosis reflect this expansion. Thus, these highly influential publications established a knowledge base representing both the field's early PCR‐based rapid diagnosis phase and its subsequent genomic expansion phase.

### 3.4 WHO Priority Pathogens, Molecular Platforms, and Resistance Markers

The distribution according to WHO priority pathogens indicates that high‐priority pathogens form the main axis of the corpus. The highest publication volume was observed for *S. aureus* (730 publications; 21,945 total citations); followed by *M. tuberculosis* (439 publications), *Enterobacterales* (389 publications), *P. aeruginosa* (309 publications), *A. baumannii* (279 publications), and *E. faecium* (250 publications). In terms of average citation load, *S. aureus, A. baumannii*, and *Enterobacterales* stood out, while growth rates over the past 5 years showed a significant increase, particularly for *Enterobacterales* (22.81%), *P. aeruginosa* (26.35%), and *Streptococcus pneumoniae* (27.79%) (Table 4).

**Table 4:** Distribution of publications by WHO priority pathogens.

WHO priority group	Pathogen/pathogen family	Publications	Total citations	Average citations	Dominant platform(s)	Growth rate over the past 5 years (%)
Critical	*Mycobacterium tuberculosis*	439	9056	20.63	WGS, conventional PCR/NAAT	7.79
Critical	*Enterobacterales*	389	8138	20.92	WGS, conventional PCR/NAAT	22.81
Critical	*Acinetobacter baumannii*	279	6394	22.92	Conventional PCR/NAAT, WGS	17.41
High	*Pseudomonas aeruginosa*	309	6230	20.16	WGS, conventional PCR/NAAT	26.35
High	*Staphylococcus aureus*	730	21,945	30.06	Conventional PCR/NAAT, WGS	12.86
High	*Enterococcus faecium*	250	4815	19.26	WGS, conventional PCR/NAAT	18.92
High	Nontyphoidal *Salmonella*	22	505	22.95	WGS, conventional PCR/NAAT	13.62
High	*Shigella* spp.	12	246	20.50	Conventional PCR/NAAT, WGS	18.92
High	*Neisseria gonorrhoeae*	8	133	16.62	WGS, conventional PCR/NAAT	NA
High	*Salmonella* Typhi	4	38	9.50	WGS, conventional PCR/NAAT	NA
Medium	*Streptococcus pneumoniae*	36	877	24.36	Conventional PCR/NAAT, multiplex PCR	27.79
Medium	Group B *Streptococcus*	23	595	25.87	Conventional PCR/NAAT, multiplex PCR	−24.02
Medium	*Haemophilus influenzae*	15	485	32.33	Conventional PCR/NAAT, multiplex PCR	NA
Medium	Group A *Streptococcus*	14	354	25.29	Conventional PCR/NAAT, multiplex PCR	18.92

*Note:* The growth rate over the last 5 years was calculated as the compound annual growth rate (CAGR) for the 2021–2025 period; cells with no record in the starting year are shown as “NA.” Platform categories are not mutually exclusive; a single publication may be assigned to more than one platform category, and the reported percentages therefore sum to more than 100%. Percentages were calculated over the total corpus (*n* = 1746).

Abbreviations: NAAT, nucleic acid amplification testing; PCR, polymerase chain reaction; WGS, whole‐genome sequencing; WHO, World Health Organization.

When examining molecular diagnostic platforms, conventional PCR/NAAT (1074 publications: 61.51%) and WGS (968 publications: 55.44%) are the two most frequently represented platform categories in the corpus. Because platform categories are not mutually exclusive, a single publication may be assigned to more than one category, and these two categories frequently co‐occur within the same publications. The high average citation count of Multiplex PCR (42.57) and the historical origins of qPCR/real‐time PCR demonstrate how pivotal early‐stage rapid diagnosis and target‐specific marker detection have been for the field. In contrast, the lower but growing visibility of platforms such as Nanopore sequencing, metagenomic sequencing, and CRISPR‐based diagnostics suggests that the field is opening up to new, high‐resolution, and portable technologies (Table 5).

**Table 5:** Distribution of publications and impact by molecular diagnostic platform.

Platform	Publications	Percentage	Total citations	Average citations	Year of first publication	Annual growth over the past 5 years (%)	Most frequent pathogen contexts in matched records	Most frequent marker contexts in matched records
Conventional PCR/NAAT	1074	61.51	30,645	28.53	2000	16.23	*Staphylococcus aureus*, *Enterobacterales*	*mecA/mecC*, *rpoB*, *blaNDM*
WGS	968	55.44	15,468	15.98	2010	13.37	*Mycobacterium tuberculosis*, *S. aureus*	*rpoB*, *mecA/mecC*, *blaNDM*
Multiplex PCR	412	23.60	17,537	42.57	2000	16.74	*S. aureus*, *Enterobacterales*	*mecA/mecC*, *vanA/vanB*, *blaNDM*
qPCR/real‐time PCR	360	20.62	8950	24.86	2000	11.07	*S. aureus*, *M. tuberculosis*	*mecA/mecC*, *rpoB*, *katG*
Nanopore sequencing	83	4.75	1091	13.14	2016	16.36	*Enterobacterales*, *S. aureus*	*blaNDM*, *mecA/mecC*, *16S rRNA*
Metagenomic sequencing	42	2.41	542	12.90	2014	27.79	*S. aureus*, *Enterobacterales*	*16S rRNA*, *mecA/mecC*, *rpoB*
CRISPR‐based diagnostics	25	1.43	463	18.52	2020	0.00	*Enterobacterales*, *Pseudomonas aeruginosa*	*blaNDM*, *blaKPC*, *rpoB*
Digital PCR	15	0.86	500	33.33	2013	−15.91	*Enterobacterales*, *S. aureus*	*mecA/mecC*, *katG*, *inhA*
LAMP/isothermal amplification	11	0.63	185	16.82	2017	−100.00	*S. aureus*, *M. tuberculosis*	*mecA/mecC*, *rpoB*, *katG*
POC molecular systems	10	0.57	95	9.50	2012	18.92	*M. tuberculosis*, *Enterobacterales*	*rpoB*, *mecA/mecC*, *blaKPC*

*Note:* Platform labels were determined by rule‐based text matching applied to titles, abstracts, and keywords. Pathogen and marker fields reflect the most frequent co‐occurrence contexts within matched records and should be interpreted as bibliometric associations rather than as exclusive biological specificity.

Abbreviations: CRISPR, clustered regularly interspaced short palindromic repeats; LAMP, loop‐mediated isothermal amplification; NAAT, nucleic acid amplification testing; PCR, polymerase chain reaction; POC, point of care; qPCR, quantitative PCR; WGS, whole‐genome sequencing.

At the resistance‐marker level, *mecA/mecC, rpoB, blaNDM, katG, and vanA/vanB* emerged as the most frequently represented molecular targets in the corpus (Table 6). Conventional PCR/NAAT and WGS constituted the dominant platform contexts across most marker groups, indicating that the resistance‐marker literature is structured around both targeted molecular detection and broader genomic characterization approaches. Markers such as *blaNDM, blaKPC, blaOXA‐48, mcr*, and *parC* showed particularly strong recent growth, whereas more established targets such as *mecA/mecC, rpoB*, and *vanA/vanB* maintained high representation across the study period. Because marker‐level analyses were derived from bibliographic text matching, these patterns are presented as corpus‐level bibliometric trends rather than as exclusive pathogen‐specific biological associations.

**Table 6:** Most frequently studied resistance markers and bibliometric trend characteristics.

Resistance marker/target	Publications	Associated platforms	Year of first appearance	Trend over the past 5 years (%)
mecA/mecC	583	Conventional PCR/NAAT, multiplex PCR	2000	5.48
rpoB	379	WGS, conventional PCR/NAAT	2000	8.67
blaNDM	295	WGS, conventional PCR/NAAT	2011	25.98
katG	248	WGS, conventional PCR/NAAT	2000	−5.67
vanA/vanB	239	WGS, conventional PCR/NAAT	2000	16.93
16S rRNA	178	Conventional PCR/NAAT, WGS	2000	−4.27
blaKPC	172	WGS, conventional PCR/NAAT	2011	24.32
inhA	142	WGS, conventional PCR/NAAT	2003	−12.42
blaVIM	112	Conventional PCR/NAAT, WGS	2011	10.67
gyrA	108	WGS, conventional PCR/NAAT	2004	44.28
blaIMP	98	Conventional PCR/NAAT, WGS	2011	4.66
blaOXA‐48	82	Conventional PCR/NAAT, WGS	2011	27.79
mcr	28	WGS, conventional PCR/NAAT	2019	36.78
parC	22	WGS, conventional PCR/NAAT	2007	49.53

*Note:* The trend over the past 5 years is reported on a CAGR basis for the 2021–2025 period.

Abbreviations: CAGR, compound annual growth rate; NAAT, nucleic acid amplification testing; PCR, polymerase chain reaction; rRNA, ribosomal RNA; WGS, whole‐genome sequencing.

### 3.5 Thematic Architecture, Clustering Structure, and Temporal Evolution

**Figure 5:**
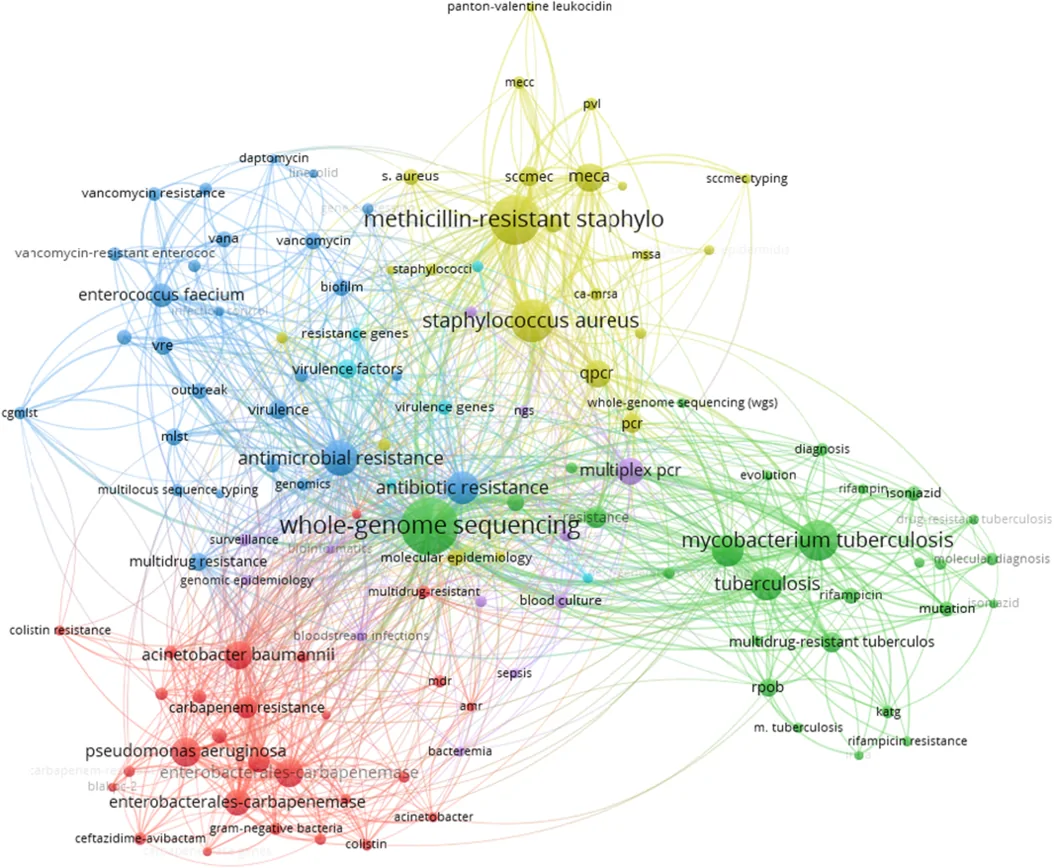
Co‐occurrence network generated from standardized keywords.

A standardized co‐occurrence network of keywords has shown that the corpus is organized around several strong thematic cores. At the center of the network are methodological and conceptual nodes with high connectivity, such as WGS, AMR, and antibiotic resistance. These terms carry the highest degree values in the co‐occurrence network; that is, they co‐occur with the largest number of distinct keywords. This structural position indicates that genomic approaches and the AMR discourse constitute the common ground linking the different thematic axes of the literature. Pathogen‐specific clusters, however, are concentrated particularly around the axes of *S. aureus*/MRSA, *M. tuberculosis, E. faecium*, and *Enterobacterales*–carbapenemase. The co‐occurrence of *mecA*, Panton–Valentine leukocidin *(PVL)*, and *SCCmec* in the staphylococcal cluster; *rpoB, katG*, and *inhA* in the tuberculosis cluster; and carbapenemases and related resistance terms in the Gram‐negative cluster indicates that the field consists of pathogen‐specific yet methodologically interconnected subresearch fronts (Figure 5).

**Figure 6:**
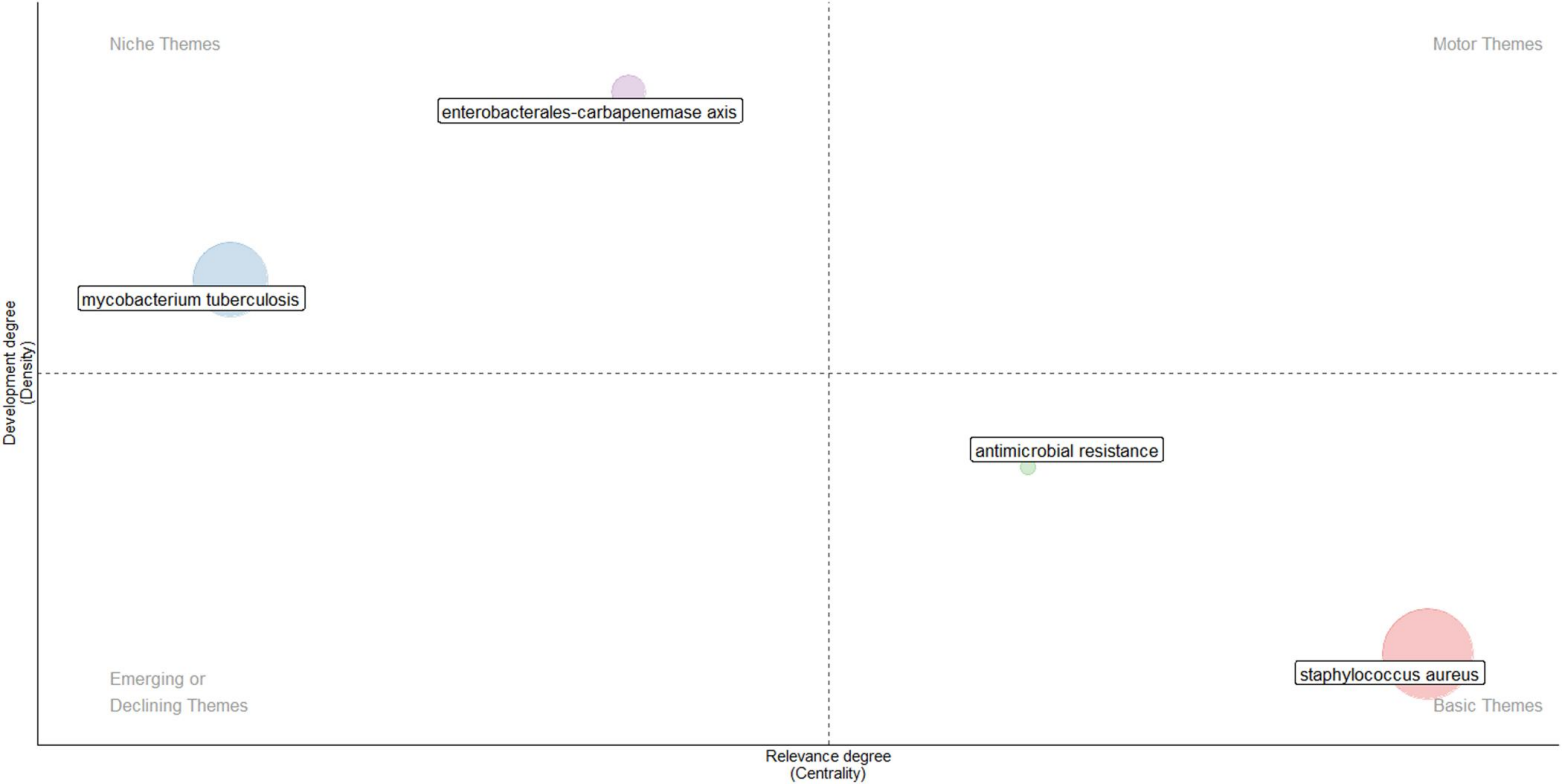
Thematic map generated after *Enterobacterales* standardization. The horizontal axis indicates centrality and the vertical axis indicates density.

The thematic map shows that the network structure is divided into layers not only by connection density but also by conceptual centrality and maturity. The positioning of the *M. tuberculosis* and *Enterobacterales*–carbapenemase axis clusters in a region of high density but relatively limited centrality suggests that these two areas represent methodologically well‐developed yet more specialized research lines. In contrast, the positioning of the *S. aureus* and AMR themes in higher‐centrality regions indicates that these axes maintain connections with a broad range of other themes on the thematic map. Centrality on the thematic map measures the degree of external connectedness of a theme and is a dimension distinct from both publication volume and citation impact (Figure 6).

The interpretive summary of the thematic clusters also confirms this structure. Rule‐based clustering identifies PCR‐based rapid AMR detection and WGS/genomic epidemiology as mature core themes. Although these two themes share the high‐frequency terms of the corpus as a whole, they diverge in platform ordering and in their discriminating marker signatures: cluster 1 is organized around multiplex PCR, is led by conventional PCR/NAAT, and represents a Gram‐positive targeted rapid‐testing theme defined by *mecA/mecC* and *vanA/vanB*, whereas cluster 2 is WGS‐led and carries a genomic characterization signature cutting across pathogen groups through the co‐occurrence of *rpoB*, *mecA/mecC*, and *blaNDM*. The tuberculosis resistance markers axis is positioned as a strong yet specialized mature theme; the carbapenemase and Gram‐negative resistance genes cluster as an emerging theme; and metagenomic/nanopore clinical diagnostics together with a general AMR‐related pathogen cluster as emerging or niche research fronts (Table 7).

**Table 7:** Thematic clusters and interpretive labels.

Cluster no.	Representative keywords	Dominant pathogen(s)	Dominant platform(s)	Dominant marker(s)	Thematic interpretation	Maturity level
1	MRSA, *Staphylococcus aureus*, antimicrobial resistance (AMR), multiplex PCR, antibiotic resistance	*S. aureus*, *Enterococcus faecium*	Conventional PCR/NAAT, WGS	*mecA*/*mecC*, *vanA*/*vanB*, *rpoB*	PCR‐based rapid AMR detection	Mature core theme
2	Whole‐genome sequencing (WGS), *Mycobacterium tuberculosis*, AMR, *S. aureus*	*S. aureus*, *M. tuberculosis*	WGS, conventional PCR/NAAT	*rpoB*, *mecA*/*mecC*, *blaNDM*	WGS and genomic epidemiology	Mature core theme
3	Nanopore sequencing, *S. aureus*, AMR, MRSA, WGS	*Enterobacterales*, *S. aureus*	Nanopore sequencing, WGS	*16S rRNA*, *blaNDM*, *mecA*/*mecC*	Metagenomic/nanopore clinical diagnostics	Emerging research frontier
4	*M. tuberculosis*, tuberculosis, drug resistance, WGS	*M. tuberculosis*, *S. aureus*	WGS, conventional PCR/NAAT	*rpoB*, *katG*, *inhA*	Tuberculosis resistance markers and molecular tests	Mature core theme
5	*Pseudomonas aeruginosa*, *Acinetobacter baumannii*, carbapenemase, WGS, AMR	*Enterobacterales*, *P. aeruginosa*	WGS, conventional PCR/NAAT	*blaNDM*, *blaKPC*, *blaVIM*	Carbapenemases and Gram‐negative resistance genes	Emerging theme
6	Tuberculosis, AMR, *M. tuberculosis*, *P. aeruginosa*, MRSA	*M. tuberculosis*, *S. aureus*	WGS, conventional PCR/NAAT	*rpoB*, *mecA*/*mecC*, *blaNDM*	General AMR‐related pathogen cluster	Niche theme

*Note:* Thematic labels were assigned through interpretive review of dominant keyword patterns within each cluster and are intended as descriptive summaries of the cluster's core rather than as fully automated or mutually exclusive topic categories. Thematic labels were assigned by interpretive review of the dominant keyword patterns within each cluster and are intended as descriptive summaries of the cluster core rather than as fully automated or mutually exclusive topic categories. The dominant pathogen, platform, and marker columns report the most frequent contexts within each cluster and are not exclusive properties of that cluster; the appearance of high‐frequency elements (e.g., WGS, *Staphylococcus aureus*, and *mecA*/*mecC*) across several clusters is therefore expected. Differentiation between clusters derives not from these shared high‐frequency elements but from cluster‐specific discriminating signatures: multiplex PCR, *vanA*/*vanB*, and a conventional PCR/NAAT‐led platform ordering for cluster 1; a WGS‐led ordering and the co‐occurrence of *rpoB* with *blaNDM* for cluster 2; nanopore sequencing and *16S rRNA* for cluster 3; *katG* and *inhA* for cluster 4; and *blaKPC* and *blaVIM* for cluster 5. Cluster 6 is distinguished instead by the absence of a single dominant pathogen focus: its representative keywords simultaneously span terms from three separate pathogen groups (*M. tuberculosis*, *P. aeruginosa*, and MRSA), a pattern consistent with its interpretation as a general, cross‐pathogen cluster and with its niche density.

Abbreviations: MRSA, Methicillin‐resistant *Staphylococcus aureus*; NAAT, nucleic acid amplification testing; PCR, polymerase chain reaction.

**Figure 7:**
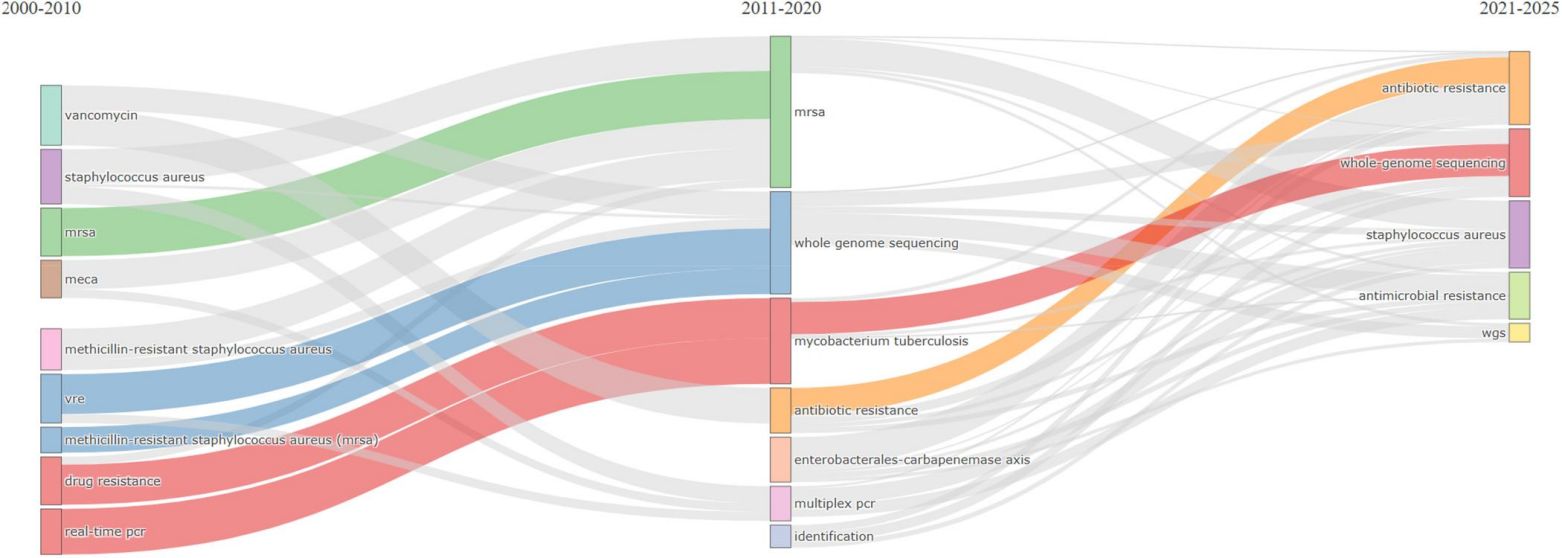
Thematic evolution diagram generated from standardized keywords.

The thematic evolution diagram reveals that the literature underwent a distinct conceptual restructuring during the 2000–2025 period. In the early period, dominant themes centered on target‐specific resistance markers, such as vancomycin, VRE, MRSA, mecA, drug resistance, and real‐time PCR, along with classical molecular amplification strategies. In the middle period, themes such as WGS, *M. tuberculosis*, antibiotic resistance, multiplex PCR, and the *Enterobacterales*–carbapenemase axis came to the fore; in the recent period, WGS, *S. aureus*, antibiotic resistance, AMR, and WGS have come to represent a more integrated AMR framework (Figure 7). This transition demonstrates that the field has evolved from an early phase focused on individual markers to a translational phase that is genomically integrated and AMR‐centered.

**Figure 8:**
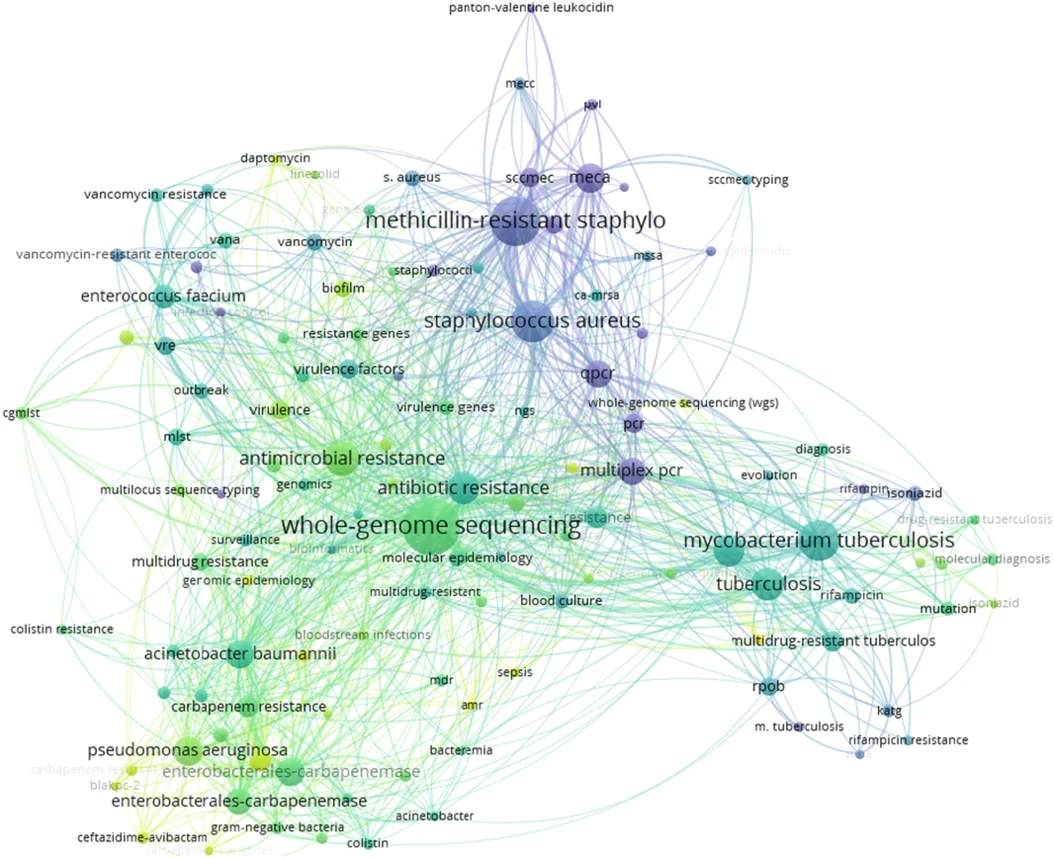
Overlay visualization of standardized keywords. Colors reflect the average publication year.

The overlay visualization has revealed the temporal distribution of themes within the co‐occurrence network in greater detail. While nodes from the earlier period were predominantly clustered around MRSA, *mecA, PVL*, and *SCCmec*; in the more recent period, concepts such as WGS, *M. tuberculosis*, *Enterobacterales*–carbapenemase, carbapenem resistance, and associated Gram‐negative resistance became prominent (Figure 8). Thus, a strong temporal consistency has been established between the co‐occurrence structure and the thematic evolution analysis; the transition from the early period's Gram‐positive resistance phenotypes and PCR‐based validation axis to the recent period's genomic resistance prediction and focus on critical Gram‐negative pathogens is clearly observable.

## 4 Discussion

In this study, a strong growth momentum was identified in the final Scopus corpus covering the 2000–2025 period, with acceleration after 2018 and, more notably, after 2020. This growth pattern suggests that AMR has gained an increasingly visible and prioritized position on the research agenda in relation to its global health burden (Murray et al. 2022; Sun et al. 2022; Ikuta et al. 2022). Similarly, previous bibliometric evaluations have also shown that the literature on antibiotic‐resistant bacteria has expanded significantly over the past decade and that the research focus has increasingly shifted toward molecular/genomic approaches and resistance‐related themes (Sun et al. 2022; Jangid et al. 2023; Shi 2025).

The dominance of the subject areas “Medicine, Immunology, and Microbiology” and “Biochemistry, Genetics, and Molecular Biology,” along with the fact that core journals consist of both clinical microbiology and AMR‐focused publications, suggests that molecular bacterial diagnostics is clearly positioned as a translational research field. This finding aligns with the broader transformation highlighted in the literature on clinical metagenomics and next‐generation sequencing; within the current framework, diagnostic approaches are shifting from culture‐dependent and single‐target methods toward hybrid workflows that integrate sequencing data, bioinformatics analysis, and standardized reporting processes (Chiu and Miller 2019; Gu et al. 2019; Kan et al. 2024). Therefore, the journal and subject‐area pattern observed in our study is significant in that it demonstrates the field is not limited to classical infection diagnosis but is expanding toward more integrated and data‐intensive clinical applications.

At the national level, while China and the United States lead in production volume, the higher rates of international coauthorship observed in countries such as the United Kingdom, France, and Germany suggest two distinct patterns of visibility within the field: volume‐based productivity versus network‐intensive collaboration. This structure aligns with the literature emphasizing that international collaboration in infectious disease research affects not only data sharing but also method harmonization, surveillance capacity, and the speed of information production during crises (Fanning et al. 2021). Additionally, bibliometric studies on AMR have reported that China and the United States stand out in terms of publication volume, whereas visible and effective collaboration networks are frequently shaped by multihub partnership structures (Sun et al. 2022). Therefore, the coauthorship network observed in our study suggests that the molecular bacterial diagnostics literature is shaped not only by national production clusters but also by international collaboration networks.

Why these publication‐volume patterns cluster around particular pathogens, markers, and platforms can be explained by the field's historical and clinical conditions. The dominance of a given pathogen–marker–platform combination reflects not only its clinical burden but also how well its target is molecularly defined, how readily it can be reduced to a standard assay target, and how easily it fits the existing diagnostic infrastructure. The early and sustained dominance of Gram‐positive resistance markers (*mecA/mecC* and *vanA/vanB*) is consistent with the fact that these targets are well defined at the single‐gene level and directly translatable into PCR targets; the establishment of the tuberculosis axis as a mature theme is consistent with the long‐standing characterization of its resistance markers (*rpoB*, *katG*, and *inhA*) and the global surveillance priority of the disease; and the emerging position of the *Enterobacterales*–carbapenemase axis is consistent with the comparatively recent but rapidly growing clinical urgency of carbapenem resistance. These patterns are examined individually in the sections that follow.

The fact that a significant portion of the most‐cited publications cluster around MRSA, *mecA/SCCmec*, and multiplex PCR indicates that Gram‐positive resistance markers have played a distinct foundational role in the field's historical development. In particular, Oliveira and de Lencastre's multiplex PCR strategy for *mec* element typing and Zhang and colleagues' *SCCmec* subtyping approach have demonstrated that standardized targets in molecular MRSA diagnosis form a robust methodological foundation (Oliveira and Lencastre 2002; Zhang et al. 2005). Modular PCR systems developed later with the inclusion of mecC have also shown how the classic mecA‐centered paradigm has been expanded to include new resistance variants (Becker et al. 2013). In this context, the fact that *S. aureus* appears as one of the most prevalent high‐priority pathogens in our study, with 730 publications and 21,945 total citations, reflects not only a volume‐based concentration but also the long‐term impact of targeted rapid molecular tests in clinical laboratory practice (Sati et al. 2025; Oliveira and Lencastre 2002; Zhang et al. 2005; Becker et al. 2013).

The fact that conventional PCR/NAAT has the highest frequency at the platform level, while WGS has gained significant prominence, indicates a complementary usage pattern that addresses different clinical needs rather than one method completely replacing the other in molecular diagnostics. Targeted PCR‐based tests still hold high practical value, particularly for the rapid detection of specific resistance markers, such as *mecA/mecC, vanA/vanB*, and core carbapenemase genes. The multiplex real‐time PCR approach developed by Monteiro et al. (2012) for carbapenemases and the study by Probst et al. (2021) comparing RT‐qPCR and WGS in *Enterobacterales* directly support this clinical rationale. In contrast, WGS has gained increasing importance in recent years, particularly for the more comprehensive characterization of resistance determinants and the enhancement of susceptibility/resistance prediction in specific pathogens (Meehan et al. 2019; Walker et al. 2015; Lam et al. 2021). In this context, when the numerical superiority of conventional PCR/NAAT is evaluated alongside the high conceptual value of WGS in our study, it becomes clear that the literature has established a functional complementarity between rapid targeted diagnosis and more comprehensive genomic analysis.

One of the most prominent examples of this dual structure is the *M. tuberculosis* literature. In our study, *M. tuberculosis* was among the most prominent axes of high‐priority pathogens, with 439 publications; the *rpoB, katG*, and *inhA* targets stood out. This pattern is consistent with the literature, indicating that while classical genotypic markers remain important in tuberculosis resistance diagnosis, WGS is assuming an increasingly central role in predicting drug susceptibility/resistance and in outbreak investigations (Meehan et al. 2019; Walker et al. 2015; Lam et al. 2021). Meehan and colleagues systematically discussed current standards and outstanding issues for WGS, while Walker and colleagues demonstrated that WGS can capture a broad spectrum of mutations for resistance prediction (Meehan et al. 2019; Walker et al. 2015). Lam and colleagues' report that routine WGS use provides added value in AMR management also offers a framework consistent with the fact that tuberculosis appears as a mature and established theme in our study (Lam et al. 2021).

One of the study's notable findings is that the *Enterobacterales*–carbapenemase axis is discussed under diagnostic method subheadings, such as phenotypic, immunochromatographic, proteomic, and molecular. In particular, the literature emphasizes that carbapenemase clusters such as *Klebsiella pneumoniae* carbapenemase (KPC), New Delhi metallo‐β‐lactamase (NDM), and OXA‐48‐type enzymes (OXA‐48) play a significant role in clinical and diagnostic decision‐making processes (Probst et al. 2021; Monteiro et al. 2012; O'toole et al. 2023; Y. L. Lee et al. 2022).

Studies comparing real‐time multiplex PCR with WGS in *Enterobacterales* have shown that PCR‐based approaches play a complementary role in rapid clinical screening and the early detection of resistance markers, while WGS serves a complementary role in more detailed genomic characterization, transmission analysis, and the elucidation of resistance architecture (Probst et al. 2021; Monteiro et al. 2012; C. R. Lee et al. 2016). Furthermore, Carbapenemase‐producing *Enterobacterales* studies focused on genomic surveillance demonstrate that genomic data in the context of *Enterobacterales* serves not only as a confirmatory tool but also as a critical public health component that reveals transmission chains (C. R. Lee et al. 2016). Therefore, the prevalence of markers such as *blaNDM, blaKPC*, and *blaOXA‐48* for *Enterobacterales* in our study is strongly consistent with the current literature on Gram‐negative resistance (Probst et al. 2021; Monteiro et al. 2012; O'toole et al. 2023; Y. L. Lee et al. 2022; C. R. Lee et al. 2016).

This carbapenemase‐centered literature, however, largely excludes the genetic background that carries the resistance determinant. The global dissemination of carbapenemase genes does not occur at random but proceeds through defined high‐risk clonal lineages; lineages such as *K. pneumoniae* ST258 and ST11, *Escherichia coli* ST131, and *P. aeruginosa* ST235 display advantages in persistence and spread within the hospital ecology that are independent of the resistance genes they carry. A comparable pattern has been reported for the VRE cluster observed along the *vanA/vanB* axis in our corpus (Sherry et al. 2019). In parallel, hypervirulent *K. pneumoniae* presents a clinical picture distinct from that of classical healthcare‐associated strains through virulence determinants such as capsular overproduction and the *rmpA/rmpA2*, *iuc*, and *iro* loci encoding the aerobactin and salmochelin systems; the convergence of carbapenem resistance and hypervirulence within a single strain gives rise to isolates that are simultaneously difficult to treat and highly capable of dissemination (Hetta et al. 2025). The diagnostic implication of this distinction is direct: marker‐targeted PCR panels detect the resistance gene but cannot resolve the clonal background in which it resides, its plasmid context, or the accompanying virulence loci; characterization at this level requires WGS, which provides resolution at the level of sequence type and virulence locus. The PCR–WGS complementarity observed in our corpus, therefore, reflects not only a difference in resolution but also a distinction in which biological question is being answered.

The persistence of the *E. faecium*/VRE axis as a smaller yet distinct cluster is also clinically significant. The fact that *vanA/vanB* markers rank among the most frequently studied targets in our study and that *E. faecium* constitutes a strong high‐priority theme with 250 publications reflects VRE's role, particularly within the ecology of hospital‐associated infections. Current literature indicates that new *E. faecium* strains can become dominant in healthcare settings and that vancomycin resistance is one of the key components of this hospital‐associated ecology (Sherry et al. 2019). In this context, the VRE cluster in our study, while not as extensive as MRSA in the literature, represents a persistent and structural research line in terms of hospital‐based AMR management (Sherry et al. 2019).

From the perspective of emerging platforms, the fact that metagenomic sequencing appears as an emerging theme despite being represented at a relatively low frequency reflects the delay between technological innovation and its integration into clinical routine. Although clinical metagenomics offers strong potential due to its broad diagnostic scope and culture‐independent pathogen detection capabilities, issues such as preanalytical standardization, contamination control, reference databases, and result reporting standards still limit its widespread applicability (Chiu and Miller 2019; Gu et al. 2019; Kan et al. 2024).

Oxford Nanopore‐based approaches provide near‐real‐time data generation and the advantage of long reads; they have yielded promising results, particularly in *M. tuberculosis* drug susceptibility testing and outbreak investigations. However, due to its error profile, bioinformatics requirements, and the need for comprehensive validation, it has not yet become a widely adopted standard platform (Sheka et al. 2021; Hall et al. 2023). Similarly, while CRISPR‐Cas‐based strategies hold strong conceptual potential for the targeted detection of resistance genes, they have not yet reached the stage of large‐scale integration into clinical diagnostic workflows (Ahmed et al. 2024). In our study, the emergence of these technologies in low‐frequency yet distinct clusters suggests they represent innovation areas that have not yet become dominant in the molecular diagnostics literature.

A holistic analysis of the thematic clusters reveals that the field is organized around three interconnected core areas: rapid, targeted AMR detection; genomic epidemiology/WGS; and resistance markers specific to high‐priority pathogens. This structure suggests that molecular diagnostics has evolved from being merely a laboratory tool that answers the “pathogen present/absent” question into a multilayered data‐generating field that supports resistance prediction, monitoring of clonal spread, and clinical/public health decision‐making. In particular, the shift from the early‐period focus on MRSA–mecA–PCR to WGS and carbapenemase‐focused Gram‐negative structures in overlay and thematic evolution outputs suggests that methodological innovation has also driven a conceptual restructuring.

The structure revealed by this map depicts a literature organized around genotypic resistance determinants; however, the failure of antibiotic therapy does not arise from genetically encoded resistance alone. Bacterial dormancy is a reversible state of reduced metabolic activity and arrested replication, governed by signaling pathways, transcriptional and translational regulation, and metabolic shifts; the persister subpopulations arising within this state can tolerate high antibiotic concentrations while carrying no resistance gene and showing no change in minimum inhibitory concentration (Abid et al. 2025). Its clinical counterparts are relapse despite apparently adequate therapy, the intractability of foreign‐body and biofilm‐associated infections, and the prolonged treatment regimens required in tuberculosis. The diagnostic consequence bears directly on our findings: both marker‐targeted PCR panels and WGS report the genetic content of a bacterial population rather than its physiological state. A fully susceptible genotype can therefore coexist with treatment failure driven by tolerance, and the capacity for resistance prediction developed by this literature remains bounded at the level of genotype. That the persistence and dormancy axis appears only marginally in the present map is due in part to the methodological nature of this field: tolerance and persistence are assessed largely through phenotypic approaches (time–kill curves, tolerance assays) that are not represented in the marker‐centered vocabulary of the molecular diagnostics literature.

### 4.1 Study Limitations

Certain limitations should be considered when interpreting the findings. Because the analysis was restricted to Scopus‐indexed articles and reviews published in English, relevant outputs from regional databases, nonindexed sources, or publications in other languages may have been excluded. The possible influence of this restriction on the findings can be considered at three levels. First, because indexing coverage is skewed toward English‐language journals, studies published in regional and national journals—particularly from low‐ and middle‐income countries—may be underrepresented in the corpus, which would tend to depress absolute publication counts in country‐level productivity rankings (Mongeon and Paul‐Hus 2016). Second, citation counts are database‐specific; the total and mean citation values reported here should therefore be read as Scopus‐derived and should not be compared directly with bibliometric studies based on Web of Science. Third, coverage differences would be expected to affect absolute magnitudes most and relative structure least: structural findings such as rank order, thematic cluster composition, and temporal trends are considered more robust to the choice of database (Mongeon and Paul‐Hus 2016; Singh et al. 2021). A multidatabase validation study would nonetheless be valuable in testing this assumption.

Citation‐based impact indicators inherently favor older and foundational publications; consequently, newer technologies such as CRISPR‐based diagnostics, clinical metagenomics, and nanopore sequencing may appear to be at a relatively early stage in terms of citation density despite their conceptual relevance and growing translational potential. In addition, although the rule‐based tagging framework showed high concordance in the validation subset, some degree of ambiguity may remain in records containing overlapping terminology, broad, multitopic abstracts, or context‐dependent mentions that do not reflect the publication's primary focus. Therefore, the derived pathogen‐, platform‐, marker‐, and clinical‐context variables should be interpreted as analytically structured bibliometric categories rather than as fully manual expert annotations. Furthermore, the fact that record screening and rule‐based classification were performed by a single researcher entails a possibility of selection bias. This risk was mitigated through the application of predefined eligibility criteria and a subset reassessment conducted blind to the automated assignments (overall mean agreement 94.5%), but it could not be eliminated entirely. Independent screening of all records by multiple reviewers would strengthen the design of future studies.

In addition, the marker vocabulary was built around resistance determinants (*mecA/mecC*, *vanA/vanB*, *blaKPC*, *blaNDM*, *blaOXA‐48*, *rpoB*, *katG*, and *inhA*) and did not include virulence loci or clonal lineage designations (sequence types, clonal complexes). Publications centered on hypervirulence and high‐risk clones could therefore enter the corpus only to the extent that they also referenced these resistance markers, and the true volume of that literature may consequently appear lower in the present map than it is. Likewise, phenotypic and physiological states such as tolerance, persistence, and dormancy fall outside the scope of the marker vocabulary. The present map, therefore, represents the genotypic resistance‐detection dimension of molecular diagnostics and does not encompass the phenotypic determinants of antibiotic response.

Nonetheless, the concurrent visibility of emerging platforms alongside ongoing challenges related to standardization, validation, and clinical integration suggests that the “low‐volume but high‐innovation” pattern observed in this study likely reflects not only bibliometric characteristics but also genuine technological maturation dynamics.

## 5 Conclusions

This bibliometric analysis demonstrates that the literature on molecular bacterial diagnostics and AMR has grown rapidly in volume between 2000 and 2025; however, this growth was not random but occurred alongside a structural reorganization centered around specific pathogens, resistance markers, and platform technologies. The study's main axes have crystallized around *S. aureus*/MRSA, *M. tuberculosis*, *Enterobacterales*–carbapenemase‐associated Gram‐negative resistance, and WGS‐based genomic epidemiology; this pattern aligns closely with the current global burden of AMR and the direction of modern clinical microbiology.

Consequently, the field is progressing through a dual phase in which classical PCR‐based targeted tests retain their clinical value, while WGS has become the most frequently represented platform in the research and surveillance literature and the most highly connected node in the keyword co‐occurrence network; metagenomic, nanopore, and CRISPR‐based approaches, though currently limited in share, are emerging as key innovation fronts for the future.

## Author Contributions

**Selahattin Ünlü:** conceptualization, investigation, writing – original draft, methodology, validation, visualization, writing – review and editing, software, formal analysis, project administration, data curation, supervision, resources.

## Funding

The author has nothing to report.

## Ethics Statement

Ethical approval was not required for this study because it was based exclusively on publicly indexed bibliographic metadata and did not involve human participants, patient‐level data, animal experiments, biological specimens, or identifiable personal information.

## Conflicts of Interest

The author declares no conflicts of interest.

## Supporting information


Supporting File 1

Supporting File 2

Supporting File 3

Supporting File 4

Supporting File 5

Supporting File 6

## Data Availability

The data used in this study were derived from the Scopus database and consist of bibliographic metadata retrieved for the defined search period and eligibility criteria. The processed data set and analysis files are available from the corresponding author upon reasonable request. Due to database licensing restrictions, raw Scopus export files may not be publicly shared in full.
