# Supplementary material for: Molecular Diagnostics for WHO Priority Bacterial Pathogens: A Bibliometric Mapping of Diagnostic Platforms, Resistance Markers, and Antimicrobial Resistance Research Trends
Source: Microbiologyopen. 2026 Aug 31;15(5):e70394. doi: 10.1002/mbo3.70394 (PMC13527535; doi:10.1002/mbo3.70394)
Supplement: Supplementary file 1 [file MBO3-15-e70394-s001.docx]

**Supplementary Table S6.** Top 20 most cited publications.

| First author | Year | Article title | Journal | Total citations | Annual normalized citations | Primary focus | Platform type | Target pathogen/marker |
| --- | --- | --- | --- | --- | --- | --- | --- | --- |
| Poirel L. | 2011 | Multiplex PCR for the detection of acquired carbapenemase genes | Diagnostic Microbiology and Infectious Disease | 1,762 | 117.47 | Carbapenemase gene detection in Gram-negative bacteria | Multiplex PCR, Conventional PCR / NAAT | *Acquired carbapenemase genes (including blaKPC-family targets)* |
| Oliveira D.C. | 2002 | Multiplex PCR strategy for rapid identification of structural types and variants of the mec element in methicillin-resistant *Staphylococcus aureus* | Antimicrobial Agents and Chemotherapy | 1,253 | 52.21 | *S.* aureus/MRSA diagnosis | Multiplex PCR, Conventional PCR / NAAT | *Staphylococcus aureus* / *mecA / mecC* |
| Zhang K. | 2005 | A novel multiplex PCR assay for the characterization and concurrent subtyping of staphylococcal cassette chromosome mec (SCCM) types I through V in methicillin-resistant *Staphylococcus aureus* | Journal of Clinical Microbiology | 1,005 | 47.86 | *S.* aureus/MRSA diagnosis | Multiplex PCR, Conventional PCR / NAAT | *Staphylococcus aureus* / *mecA / mecC* |
| Mehrotra M. | 2000 | Multiplex PCR for the detection of genes encoding *Staphylococcus aureus* enterotoxins, exfoliative toxins, toxic shock syndrome toxin 1, and methicillin resistance | Journal of Clinical Microbiology | 680 | 26.15 | *S.* aureus/MRSA diagnosis | Multiplex PCR, Conventional PCR / NAAT | *Staphylococcus aureus* / *mecA / mecC* |
| Strommenger B. | 2003 | Multiplex PCR assay for the simultaneous detection of nine clinically relevant antibiotic resistance genes in *Staphylococcus aureus* | Journal of Clinical Microbiology | 534 | 23.22 | *S.* aureus/MRSA diagnosis | Multiplex PCR, Conventional PCR / NAAT | *Staphylococcus aureus* / *mecA / mecC* |
| Milheirico C. | 2007 | Update on the multiplex PCR strategies for assigning mec element types in *Staphylococcus aureus* | Antimicrobial Agents and Chemotherapy | 453 | 23.84 | *S.* aureus/MRSA diagnosis | Multiplex PCR, Conventional PCR / NAAT | *Staphylococcus aureus* |
| Perreten V. | 2010 | Clonal spread of methicillin-resistant *Staphylococcus pseudintermedius* in Europe and North America: An international multicenter study | Journal of Antimicrobial Chemotherapy | 432 | 27 | Methicillin-resistance epidemiology and clonal spread in *staphylococci* | Conventional PCR/NAAT | *Staphylococcus pseudintermedius / mecA* |
| Martineau F. | 2000 | Correlation between the resistance genotype determined by multiplex PCR assays and the antibiotic susceptibility patterns of *Staphylococcus aureus* and Staphylococcus epidermidis | Antimicrobial Agents and Chemotherapy | 387 | 14.88 | *S.* aureus/MRSA diagnosis | Multiplex PCR, Conventional PCR / NAAT | *Staphylococcus aureus* / *mecA / mecC* |
| Stegger M. | 2012 | Rapid detection, differentiation, and typing of methicillin-resistant *Staphylococcus aureus* harboring either mecA or the new mecA homologue mecALGA251 | Clinical Microbiology and Infection | 377 | 26.93 | *S.* aureus/MRSA diagnosis | Multiplex PCR, Conventional PCR / NAAT | *Staphylococcus aureus* / *mecA / mecC* |
| Huletsky A. | 2004 | New Real-Time PCR Assay for Rapid Detection of Methicillin-Resistant *Staphylococcus aureus* Directly from Specimens Containing a Mixture of Staphylococci | Journal of Clinical Microbiology | 370 | 16.82 | *S.* aureus/MRSA diagnosis | qPCR / real-time PCR, multiplex PCR | *Staphylococcus aureus* / *mecA / mecC* |
| McClure J.-A. | 2006 | Novel multiplex PCR assay for the detection of the staphylococcal virulencemarker Panton-Valentine leukocidin genes and simultaneous discrimination of methicillin-susceptible from methicillin-resistant staphylococci | Journal of Clinical Microbiology | 355 | 17.75 | *S.* aureus/MRSA diagnosis | Multiplex PCR, Conventional PCR / NAAT | *Staphylococcus aureus* / *mecA / mecC* |
| Hillemann D. | 2007 | Evaluation of the GenoType MTBDRplus assay for rifampin and isoniazid susceptibility testing of *Mycobacterium tuberculosis* strains and clinical specimens | Journal of Clinical Microbiology | 320 | 16.84 | Tuberculosis and resistance diagnosis | qPCR / real-time PCR, Conventional PCR / NAAT | *Mycobacterium tuberculosis* / *rpoB* |
| Zhang K. | 2004 | New quadriplex PCR assay for detection of methicillin and mupirocin resistance and simultaneous discrimination of *Staphylococcus aureus* from coagulase-negative staphylococci | Journal of Clinical Microbiology | 319 | 14.5 | *S.* aureus/MRSA diagnosis | Multiplex PCR, Conventional PCR / NAAT | *Staphylococcus aureus* / *mecA / mecC* |
| Kariyama R. | 2000 | A simple and reliable multiplex PCR assay for surveillance isolates of vancomycin-resistant enterococci | Journal of Clinical Microbiology | 295 | 11.35 | VRE/E. faecium diagnosis | Multiplex PCR, Conventional PCR / NAAT | *Enterococcus faecium* / *vanA / vanB* |
| Seifert M. | 2015 | Genetic mutations associated with isoniazid resistance in *Mycobacterium tuberculosis*: A systematic review | PLoS ONE | 273 | 24.82 | Isoniazid-resistance mutations in *Mycobacterium tuberculosis* | Systematic review of molecular resistance markers | *Mycobacterium tuberculosis* / *katG* |
| Depardieu F. | 2004 | Detection of the van alphabet and identification of enterococci and staphylococci at the species levels by multiplex PCR | Journal of Clinical Microbiology | 266 | 12.09 | Van-gene detection and species-level identification of *enterococci and staphylococci* | Multiplex PCR, Conventional PCR / NAAT | *Enterococcus faecium* / *vanA / vanB* |
| Harkins C.P. | 2017 | Methicillin-resistant *Staphylococcus aureus* emerged long before the introduction of methicillin into clinical practice | Genome Biology | 263 | 29.22 | *S.* aureus/MRSA diagnosis | WGS | *Staphylococcus aureus* / *mecA / mecC* |
| Leopold S.R. | 2014 | Bacterial whole-genome sequencing revisited: Portable, scalable, and standardized analysis for typing and detection of virulence and antibiotic resistance genes | Journal of Clinical Microbiology | 260 | 21.67 | WGS-based bacterial typing and resistance/virulence gene detection | WGS | Bacterial virulence and antibiotic resistance genes |
| Kaya H. | 2018 | SCCmecFinder, a web-based tool for typing the staphylococcal cassette chromosome mec in *Staphylococcus aureus* using whole-genome sequence data | mSphere | 255 | 31.88 | WGS-based SCCmec typing in *Staphylococcus aureus* | WGS | *Staphylococcus aureus / SCCmec, mecA, mecC* |
| Cohen K.A. | 2015 | Evolution of Extensively Drug-Resistant Tuberculosis over Four Decades: Whole Genome Sequencing and Dating Analysis of *Mycobacterium tuberculosis* Isolates from KwaZulu-Natal | PLoS Medicine | 238 | 21.64 | Tuberculosis and resistance diagnosis | WGS | *Mycobacterium tuberculosis* / *rpoB* |

Note. Annual normalized citations were calculated by dividing total citations by the number of years for which the publication was visible within the dataset.
