# Supplementary material for: Molecular Diagnostics for WHO Priority Bacterial Pathogens: A Bibliometric Mapping of Diagnostic Platforms, Resistance Markers, and Antimicrobial Resistance Research Trends
Source: Microbiologyopen. 2026 Aug 31;15(5):e70394. doi: 10.1002/mbo3.70394 (PMC13527535; doi:10.1002/mbo3.70394)
Supplement: Supplementary file 2 [file MBO3-15-e70394-s002.docx]

**Supplementary Table S3.** Most productive authors.

| Rank | Author | Publications | Total citations | Average citations | h-index | Year of first publication | Last publication year |
| --- | --- | --- | --- | --- | --- | --- | --- |
| 1 | Yu Y. | 21 | 314 | 14.95 | 10 | 2021 | 2025 |
| 2 | Clark T.G. | 19 | 535 | 28.16 | 11 | 2014 | 2025 |
| 3 | Parkhill J. | 17 | 930 | 54.71 | 13 | 2014 | 2024 |
| 4 | Phelan J.E. | 17 | 455 | 26.76 | 10 | 2016 | 2025 |
| 5 | Howden B.P. | 15 | 378 | 25.2 | 11 | 2010 | 2025 |
| 6 | Ehricht R. | 15 | 125 | 8.33 | 6 | 2017 | 2025 |
| 7 | Peacock S.J. | 14 | 543 | 38.79 | 11 | 2014 | 2025 |
| 8 | Zhao Y. | 14 | 264 | 18.86 | 9 | 2015 | 2025 |
| 9 | Monecke S. | 14 | 120 | 8.57 | 6 | 2017 | 2025 |
| 10 | Niemann S. | 13 | 676 | 52 | 9 | 2004 | 2025 |
