# Supplementary material for: Molecular Diagnostics for WHO Priority Bacterial Pathogens: A Bibliometric Mapping of Diagnostic Platforms, Resistance Markers, and Antimicrobial Resistance Research Trends
Source: Microbiologyopen. 2026 Aug 31;15(5):e70394. doi: 10.1002/mbo3.70394 (PMC13527535; doi:10.1002/mbo3.70394)
Supplement: Supplementary file 3 [file MBO3-15-e70394-s003.docx]

**Supplementary Table S4.** Most productive institutions.

| Rank | Institution | Publications | Total citations | Average citations | h-index | Year of first publication | Last publication year |
| --- | --- | --- | --- | --- | --- | --- | --- |
| 1 | Zhejiang University School of Medicine | 28 | 419 | 14.96 | 12 | 2021 | 2025 |
| 2 | Islamic Azad University | 28 | 180 | 6.43 | 8 | 2010 | 2025 |
| 3 | University of Cambridge | 27 | 1,257 | 46.56 | 16 | 2012 | 2025 |
| 4 | London School of Hygiene and Tropical Medicine | 23 | 769 | 33.43 | 13 | 2014 | 2025 |
| 5 | Tehran University of Medical Sciences | 21 | 427 | 20.33 | 12 | 2008 | 2024 |
| 6 | Zhejiang University | 21 | 399 | 19 | 11 | 2021 | 2025 |
| 7 | Chinese Center for Disease Control and Prevention | 21 | 307 | 14.62 | 11 | 2014 | 2025 |
| 8 | Statens Serum Institut | 19 | 1058 | 55.68 | 11 | 2006 | 2025 |
| 9 | Mahidol University | 19 | 440 | 23.16 | 13 | 2008 | 2025 |
| 10 | University of Copenhagen | 18 | 969 | 53.83 | 10 | 2010 | 2025 |
