# Supplementary material for: Molecular Diagnostics for WHO Priority Bacterial Pathogens: A Bibliometric Mapping of Diagnostic Platforms, Resistance Markers, and Antimicrobial Resistance Research Trends
Source: Microbiologyopen. 2026 Aug 31;15(5):e70394. doi: 10.1002/mbo3.70394 (PMC13527535; doi:10.1002/mbo3.70394)
Supplement: Supplementary file 4 [file MBO3-15-e70394-s004.docx]

**Supplementary Table S1.** Scopus Boolean search query

TITLE-ABS-KEY ( ( "Acinetobacter baumannii" OR "Pseudomonas aeruginosa" OR "Staphylococcus aureus" OR "Neisseria gonorrhoeae" OR "Enterococcus faecium" OR "Mycobacterium tuberculosis" ) AND ( "real-time PCR" OR "multiplex PCR" OR "digital PCR" OR "whole genome sequencing" OR "metagenomic sequencing" OR "nanopore sequencing" OR "CRISPR-based detection" ) AND ( mecA OR vanA OR vanB OR blaKPC OR blaNDM OR blaOXA-48 OR rpoB OR katG OR inhA OR "pathogen identification" OR "genotypic resistance" ) ) AND NOT TITLE-ABS-KEY ( fung* OR virus OR parasit* OR wastewater OR soil OR poultry OR swine OR cattle ) AND ( LIMIT-TO ( DOCTYPE , "ar" ) OR LIMIT-TO ( DOCTYPE , "re" ) ) AND ( LIMIT-TO ( LANGUAGE , "English" ) ) AND PUBYEAR > 2000 AND PUBYEAR < 2025
