# Supplementary material for: Molecular Diagnostics for WHO Priority Bacterial Pathogens: A Bibliometric Mapping of Diagnostic Platforms, Resistance Markers, and Antimicrobial Resistance Research Trends
Source: Microbiologyopen. 2026 Aug 31;15(5):e70394. doi: 10.1002/mbo3.70394 (PMC13527535; doi:10.1002/mbo3.70394)
Supplement: Supplementary file 5 [file MBO3-15-e70394-s005.docx]

**Supplementary Table S2.** Most productive and most influential journals.

| Journal title | Publications | Total citations | Average citations per article | h-index | 2024 SJR quartile | First publication year | Last publication year |
| --- | --- | --- | --- | --- | --- | --- | --- |
| Journal of Clinical Microbiology | 98 | 9,202 | 93.9 | 50 | Q1 | 2000 | 2025 |
| Frontiers in Microbiology | 93 | 1,535 | 16.51 | 22 | Q1 | 2016 | 2025 |
| Antibiotics | 82 | 704 | 8.59 | 15 | Q1 | 2020 | 2025 |
| Microbiology Spectrum | 64 | 540 | 8.44 | 13 | Q1 | 2021 | 2025 |
| Journal of Global Antimicrobial Resistance | 49 | 468 | 9.55 | 11 | Q2 | 2016 | 2025 |
| Journal of Antimicrobial Chemotherapy | 46 | 1,884 | 40.96 | 21 | Q1 | 2000 | 2025 |
| Infection and Drug Resistance | 42 | 475 | 11.31 | 14 | Q2 | 2017 | 2025 |
| Frontiers in Cellular and Infection Microbiology | 40 | 417 | 10.42 | 13 | Q1 | 2019 | 2025 |
| Antimicrobial Agents and Chemotherapy | 38 | 3,271 | 86.08 | 24 | Q1 | 2000 | 2025 |
| PLoS ONE | 37 | 1,391 | 37.59 | 23 | Q1 | 2010 | 2025 |

Note. The quartile column reports the 2024 SJR quartile.
