# Supplementary material for: Molecular Diagnostics for WHO Priority Bacterial Pathogens: A Bibliometric Mapping of Diagnostic Platforms, Resistance Markers, and Antimicrobial Resistance Research Trends
Source: Microbiologyopen. 2026 Aug 31;15(5):e70394. doi: 10.1002/mbo3.70394 (PMC13527535; doi:10.1002/mbo3.70394)
Supplement: Supplementary file 6 [file MBO3-15-e70394-s006.docx]

**Supplementary Table S5.** Most productive countries.

| Rank | Country | Publications | Total citations | Average citations | h-index | SCP | MCP | MCP/SCP ratio | Year of first publication | Year of last publication |
| --- | --- | --- | --- | --- | --- | --- | --- | --- | --- | --- |
| 1 | China | 263 | 3,648 | 13.87 | 31 | 207 | 56 | 0.27 | 2003 | 2025 |
| 2 | United States | 252 | 9,042 | 35.88 | 46 | 102 | 150 | 1.47 | 2001 | 2025 |
| 3 | United Kingdom | 130 | 5,833 | 44.87 | 33 | 24 | 106 | 4.42 | 2003 | 2025 |
| 4 | Germany | 127 | 4,580 | 36.06 | 34 | 51 | 76 | 1.49 | 2003 | 2025 |
| 5 | India | 118 | 2,002 | 16.97 | 23 | 86 | 32 | 0.37 | 2002 | 2025 |
| 6 | Iran | 109 | 1,849 | 16.96 | 23 | 99 | 10 | 0.10 | 2008 | 2025 |
| 7 | Australia | 84 | 2,342 | 27.88 | 27 | 37 | 47 | 1.27 | 2002 | 2025 |
| 8 | France | 80 | 4,386 | 54.82 | 26 | 26 | 54 | 2.08 | 2004 | 2025 |
| 9 | Brazil | 74 | 1,208 | 16.32 | 18 | 48 | 26 | 0.54 | 2003 | 2025 |
| 10 | Japan | 66 | 1,406 | 21.30 | 20 | 37 | 29 | 0.78 | 2000 | 2025 |

SCP: Single-Country Publications; MCP: Multi-Country Publications.
